# Supplementary material for: Very early invasive angiography versus standard of care in higher-risk non-ST elevation myocardial infarction: study protocol for the prospective multicentre randomised controlled RAPID N-STEMI trial
Source: BMJ Open. 2022 May 3;12(5):e055878. doi: 10.1136/bmjopen-2021-055878 (PMC9066091; doi:10.1136/bmjopen-2021-055878)
Supplement: Author's manuscript [file bmjopen-2021-055878.draft_revisions.pdf]

BMJ Open is committed to open peer review. As part of this commitment we make the peer review history of every article we publish publicly available.

When an article is published we post the peer reviewers' comments and the authors' responses online. We also post the versions of the paper that were used during peer review. These are the versions that the peer review comments apply to.

The versions of the paper that follow are the versions that were submitted during the peer review process. They are not the versions of record or the final published versions. They should not be cited or distributed as the published version of this manuscript.

BMJ Open is an open access journal and the full, final, typeset and author-corrected version of record of the manuscript is available on our site with no access controls, subscription charges or pay-per-view fees (http://bmjopen.bmj.com).

If you have any questions on BMJ Open's open peer review process please email info.bmjopen@bmj.com

BMJ Open

A prospective multicentre randomised-controlled trial testing very early invasive angiography versus standard of care in higher-risk non-ST elevation myocardial infarction: rationale and design of the RAPID N-STEMI study

Journal:	*BMJ Open*
Manuscript ID	bmjopen-2021-055878
Article Type:	Protocol
Date Submitted by the Author:	26-Jul-2021

Complete List of Authors:	Kite, Thomas; Glenfield Hospital, Department of Cardiovascular Sciences<br>Banning, Amerjeet S.; Glenfield Hospital, Department of Cardiovascular Sciences<br>Ladwiniec, Andrew<br>Gale, Chris; University of Leeds<br>Greenwood, John; Leeds Teaching Hospitals NHS Trust, Cardiology; University of Leeds, Biomedical Imaging Sciences<br>Dalby, Miles; Royal Brompton & Harefield NHS Foundation Trust, Imperial College London; Royal Brompton & Harefield NHS Foundation Trust<br>Hobson, Rachel; University of Leicester<br>Barber, Shaun; University of Leicester<br>Parker, Emma; Glenfield Hospital, Department of Cardiovascular Sciences<br>Berry, Colin; University of Glasgow, British Heart Foundation Glasgow Cardiovascular Research Centre; University of Glasgow<br>Flather, Marcus; University of East Anglia, Norwich Medical School<br>Curzen, Nick; University Hospital Southampton NHS F Trust, Wessex Cardiac Unit<br>Banning, AP; Oxford University Hospitals NHS Foundation Trust, Cardiology Department, Oxford Heart Centre<br>McCann, Gerry; Glenfield Hospital, Department of Cardiovascular Sciences<br>GERSHLICK, ANTHONY; University Hospitals of Leicester, Cardiology

Keywords:	Ischaemic heart disease < CARDIOLOGY, Myocardial infarction < CARDIOLOGY, Coronary intervention < CARDIOLOGY, Coronary heart disease < CARDIOLOGY

SCHOLARONE™
Manuscripts

A prospective multicentre randomised-controlled trial testing very early invasive angiography versus standard of care in higher-risk non-ST elevation myocardial infarction: rationale and design of the RAPID N-STEMI study

Thomas A. Kite BMedSci (Hons) BM BS MRCP[1], Amerjeet S. Banning MBBS PhD MRCP[1], Andrew Ladwiniec MA MD FRCP[1], Chris P. Gale BSc PhD FRCP[2], John P. Greenwood MBChB PhD FRCP[2], Miles Dalby BSc MBBS MD[3], Rachel Hobson BA(Hons) [4], Shaun Barber BSc MSc PhD[4], Emma Parker[1], Colin Berry MBChB PhD[5], Marcus Flather MBBS MBA FRCP[6], Nick Curzen BM (Hons) PhD[7], Adrian P. Banning MB BS, MD, FRCP[8], Gerry P. McCann[1], Anthony H. Gershlick BSc MB BS FRCP[1]

Affiliations:

1. Department of Cardiovascular Sciences, University of Leicester and the Leicester Biomedical Research Centre, Glenfield Hospital, Leicester, UK
2. Leeds Institute of Cardiovascular and Metabolic Medicine, University of Leeds and the Department of Cardiology Leeds Teaching Hospitals NHS Trust, Leeds, UK
3. Royal Brompton and Harefield NHS Foundation Trust, London, UK
4. Leicester Clinical Trials Unit, University of Leicester, University Road, Leicester, UK
5. Institute of Cardiovascular and Medical Sciences, University of Glasgow, Glasgow, UK
6. Norwich Medical School, University of East Anglia, Norwich, UK
7. Faculty of Medicine, University of Southampton and University Hospital Southampton NHS Trust Southampton, UK
8. John Radcliffe Hospital, Oxford University Hospitals NHS Trust, Oxford, UK

Corresponding author:
Dr Thomas A. Kite
Department of Cardiovascular Sciences, University of Leicester and the Leicester Biomedical Research Centre, Glenfield Hospital, Leicester, LE3 9QP, UK
Email: tom.kite@nhs.net

Abstract

Introduction: There are a paucity of randomised data on the optimal timing of invasive coronary angiography (ICA) in higher-risk patients with non-ST elevation myocardial infarction (N-STEMI). International guideline recommendations for early ICA are primarily based on retrospective subgroup analyses of neutral trials. The RAPID N-STEMI trial aims to determine whether very early percutaneous revascularisation improves clinical outcomes as compared to a standard of care strategy in higher-risk N-STEMI patients.

Methods and analysis: RAPID N-STEMI is a prospective, multi-centre, open-label, randomised-controlled, pragmatic strategy trial. Higher-risk N-STEMI patients, as defined by Global Registry of Acute Coronary Events (GRACE) 2.0 score ≥118, or >90 with at least one additional high-risk feature, were randomised to either: very early ICA +/- revascularisation or standard of care timing of ICA +/- revascularisation. The primary endpoint is a composite of all-cause mortality, non-fatal myocardial infarction, and hospital admission for heart failure at 12 months. Key secondary outcomes include major bleeding and stroke. A hypothesis generating cardiac magnetic resonance (CMR) substudy will provide mechanistic data on infarct size, myocardial salvage, and residual ischaemia post percutaneous coronary intervention. On 7th April 2021, the sponsor discontinued enrolment due to the impact of the COVID-19 pandemic and lower than expected event rates. 425 patients were enrolled, and 61 patients underwent CMR.

Summary: The optimal timing of revascularisation in higher-risk N-STEMI remains controversial. RAPID N-STEMI will provide insights into the impact of very early revascularisation in GRACE score defined high-risk N-STEMI patients and inform contemporary practice in this important patient cohort.

Ethics and dissemination: The study has full ethical approval. Data collection will be completed in December 2021. The study results will be submitted for publication within 6 months of completion

Trial registration number
NCT03707314

Clinical trial registration
NCT03707314

Protocol version
v4.0

Keywords
Non-ST elevation myocardial infarction; percutaneous coronary intervention; timing; invasive strategy; GRACE score

Word count
2807

Strength and limitations of this study

- Prior data from subgroup analyses suggest that an early invasive strategy may improve clinical outcomes in high-risk patients with N-STEMI.
- RAPID N-STEMI is the first randomised-controlled trial to specifically investigate the impact of very early angiography and revascularisation in a high-risk N-STEMI population as defined by GRACE score.
- The RAPID N-STEMI cardiac magnetic resonance substudy will provide mechanistic insights into the effect of timing of revascularisation on myocardial injury and other novel cardiac magnetic resonance imaging markers.
- Recruitment to the study was discontinued prematurely due to the impact of the COVID-19 pandemic and lower than expected event rates.

Introduction

Historical data indicate that an invasive strategy during index hospital admission in non-ST elevation myocardial infarction (N-STEMI) improves composite ischaemic outcomes, with maximal benefit seen in those at highest baseline risk for future major adverse cardiovascular events (MACE).[1] However, the optimal timing of invasive coronary angiography (ICA) and whether high-risk subgroups should be treated early remains controversial, despite it being a mandated management strategy in international guidelines.[2 3] Since N-STEMI is characterised by a risk-treatment paradox whereby higher-risk patients are less likely to receive aggressive pharmacotherapy and invasive management,[4] use and timing of ICA can differ significantly when compared to the precisely defined management of ST elevation myocardial infarction (STEMI). Clinically unstable patients require urgent revascularisation, whilst for others, the most appropriate timing of an invasive strategy is less certain.

A series of randomised trials have investigated this concept in unselected N-STEMI populations, thereby testing whether early revascularisation (<24 hours) improves clinical outcomes as compared to delayed or standard of care approaches.[5-18] Differences in study design, inclusion criteria, timing of ICA and endpoint definitions have resulted in conflicting results that are challenging to interpret and apply to current practice. When these data are evaluated in totality, patient level meta-analysis has demonstrated no significant difference in death or myocardial infarction (MI) between the two strategies.[19]

The application of the Global Registry of Acute Coronary Events (GRACE) score in prior trial subgroup analyses has potential for risk stratification of those patients that may benefit most from expeditious revascularisation (**Table 1**). A GRACE score >140 analysis of 961 patients

from the TIMACS trial showed that an early invasive strategy (14 hours post randomisation) reduced the risk of death, non-fatal MI, and stroke at 6 months by 35% as compared to a delayed approach.[9] Moreover, the VERDICT study produced a similar finding, albeit in a subgroup of 1025 GRACE >140 N-STEMI patients who underwent a very early invasive strategy (4.7 hours post randomisation).[15] However, such analyses should only be considered hypothesis generating since; 1) the primary outcome in both overall trial populations was neutral, and 2) the studies were undertaken in the era of conventional troponin and CK-MB, with up to one-quarter of patients exhibiting no biomarker rise.[9]

Given that currently available data are inconsistent and of insufficient scientific quality to inform best practice, a contemporary trial that prospectively investigates the timing of revascularisation in GRACE score defined high-risk N-STEMI is required to confirm or refute these prior observations.

Methods and analysis

Study design and inclusion criteria

The RAPID N-STEMI trial enrolled patients across 32 hospitals with on-site cardiac catheter laboratories in the United Kingdom (UK). Potential participants who experienced symptoms within 12 hours prior to admission were assessed on attendance to hospital and the research team alerted if a diagnosis of N-STEMI was suspected. N-STEMI was defined as: 1) the presence of cardiovascular symptoms suggestive of myocardial ischaemia and, 2) elevation in high-sensitivity troponin (hs-Tn) I or T. Risk stratification using the GRACE 2.0 score was then performed. Patients in whom the GRACE 2.0 score was ≥118, or ≥90 with at least one

additional feature of high-risk presentation were deemed as higher risk. The full inclusion and exclusion criteria are listed in **Table 2.**

Patients were enrolled after obtaining verbal consent once eligibility was confirmed in the Emergency Department or appropriate receiving unit. Participants were then randomised in a 1:1 fashion to either: Group A) very early ICA with a view to revascularisation; or Group B) standard of care timing of ICA with a view to revascularisation. Research team members had 6 hours from hospital admission to randomise verbally consented patients who met all eligibility criteria (**Figure 1**).

Study procedures

Randomisation was performed via either a secure centralised web-based or telephone assisted system provided by http://www.sealedenvelope.com. Those assigned to very early angiography were transferred to the cardiac catheter laboratory as soon as possible. Teams were encouraged, but not mandated to achieve a randomisation to vascular sheath insertion time of less than 90 minutes. Timing of standard of care ICA was according to typical practice at individual UK centres but encouraged to be within 72 hours of admission. Percutaneous coronary intervention (PCI) and coronary artery bypass grafting (CABG) were performed according to current international guidelines.[20] Requirement for multi-vessel revascularisation to a non-infarct related artery was at the individual operator's discretion. Optimal medical therapy, including the use of anti-platelet agents, was in accordance with current clinical guidelines.[2] Drug eluting stents were used in all cases unless there were clear contraindications. As this was a pragmatic strategy trial, all procedures performed were part of guideline directed standard of care for N-STEMI.

Patients were invited to provide verbal consent to participate in the study after being read an abridged consent form prior to randomisation. This was to avoid delay in those participants that were subsequently allocated to a very early invasive strategy. In addition to the baseline hs-Tn required for study inclusion, samples were obtained post-angiography in both trial arms and prior to angiography in the standard of care arm. EQ-5D-5L and Seattle Angina Questionnaires to assess quality of life were completed after angiography in both arms.[21 22] All patients were then asked to provide written informed consent for continuation in the study prior to hospital discharge. Study follow up visits via telephone occurred at 30 days, 6 months, and 12 months from randomisation. Clinical event reporting, EQ-5D-5L and Seattle Angina Questionnaire completion were performed at each of these timepoints.

The GRACE 2.0 score

Previous high-risk subgroup analyses utilised the original GRACE score, with a score of >140 stratifying those patients at highest baseline risk. In the TIMACS and VERDICT trials it was these groups that benefited from an early invasive strategy.[9 15] However, the updated GRACE 2.0 score demonstrates equivalent performance and is easier to implement in clinical practice as compared to the original GRACE Score.[23] A notable advantage of GRACE 2.0 is that Killip Class and serum creatinine values are not required for risk calculation. This allows rapid stratification very early during hospital admission once a hs-Tn result is available, thus obviating the need to wait for renal biochemistry results.

Patients at intermediate risk (GRACE 2.0 score ≥90 to <118) were included to attenuate the perceived age bias of the GRACE score, thereby allowing enrolment of younger patients

recognised to be at higher risk of future MACE. The following features: anterior ECG changes, ST segment depression, diabetes mellitus on medication, and hs-Tn elevation three times the upper limit of normal, have been demonstrated as determinants of poorer prognosis in N-STEMI.[24-26]

Study endpoints

The RAPID-NSTEMI trial will evaluate the effect of a very early invasive strategy on the composite primary endpoint of all-cause mortality, non-fatal MI, and admission for heart failure (HF) at 12 months following randomisation. The primary and secondary endpoints are listed in **Table 3.**

Sample size calculation and statistical analysis

RAPID N-STEMI is a superiority trial powered to detect a 25% risk reduction in the primary endpoint. With a two-sided test of α=0.05 and 80% power, 964 patients were required in each arm of the study. Assuming up to 5% withdrawal, 5% crossover and 8% requiring coronary artery bypass grafting, 1157 patients were planned to be recruited to each group, resulting in a recruitment target of 2314.

Sample size calculations were based primarily on the subgroup analysis of GRACE >140 high-risk patients in the TIMACS study. The composite endpoint of death, non-fatal MI, and stroke at 6 months occurred in 21.0% of patients in the standard care arm.[9] We decided to include admission with HF since there is evidence of this being an important outcome following N-STEMI hospitalisation. Data from *Kaul et al* show that at 12 months following N-STEMI the incidence of admission with HF was 14.8%.[27] Based on these data and use of the GRACE 2.0

score, the standard care arm composite event rate of all-cause mortality, non-fatal MI, and admission for HF in RAPID N-STEMI was estimated to be 19% at 12 months.

The CMR substudy is an exploratory mechanistic substudy that had a recruitment target of 200 patients. No formal power calculations were undertaken.

RAPID N-STEMI CMR substudy

Imaging studies confined to N-STEMI are few and primarily descriptive, documenting smaller infarct size than in STEMI.[28] Novel cardiac magnetic resonance (CMR) markers (such as myocardial strain and salvage) may add incremental prognostic information to recognised predictors such as left ventricular ejection fraction (LVEF).[29 30]

The RAPID N-STEMI CMR substudy applied multi-parametric CMR imaging to; 1) assess the impact of the timing of revascularisation on infarct size, volumes and LVEF, myocardial strain, myocardial salvage, and extracellular volume and 2) quantify ischaemic burden post-PCI to ascertain whether this can predict risk of future MACE. The RAPID N-STEMI CMR substudy endpoints are listed in **Table 4**.

Four centres with an established record of high-quality CMR research participated in the substudy. As myocardial injury and infarct size reduces early following MI,[31] timing of CMR was standardised and performed at 7 (+/-3) days post admission. This also ensured angiography +/- PCI had been undertaken in both groups, as PCI itself may be associated with further myocardial injury.[32 33] The protocol included cine imaging in long and short axes. Adenosine stress perfusion was performed to assess for residual ischaemic burden and a

gadolinium-based contrast agent administered to allow detection of myocardial necrosis and microvascular obstruction.[33] Where available, pre- and post-contrast T1 mapping sequences will facilitate estimation of extracellular volume that may indicate more subtle changes in myocardial architecture.[34]

All CMR images will be sent to the core laboratory at the National Institute for Health Research Biomedical Research Centre in Leicester for quality control and central analysis, with the interpreting clinicians blinded to patient information and allocated group.

Patient and public involvement (PPI)

The University of Leicester and the Leicester NIHR Biomedical Research Centre have a very active PPI group in cardiovascular sciences. The study outline was presented to our PPI groups for feedback prior to designing the study protocol. Furthermore, the Trial Steering Committee has a lay member for study oversight. All study participants and members of the public will be invited to return for a lecture disseminating the study's key findings on completion.

Trial coordination

Trial coordination is provided by the Leicester Clinical Trials Unit (LCTU) in collaboration with the Chief Investigator (CI) and the Trial Management Group. LCTU is responsible for overall trial conduct including data management, quality assurance and statistical reporting. LCTU undertook site initiation visits, database training, and ensures all aspects of the trial are performed to the highest ethical and research standards. The study is overseen by a Trial Steering Committee consisting of three experienced clinicians, the CI, and a lay member. An independent Data and Safety Monitoring Board convened to provide independent advice on

study conduct and safety issues. Clinical events will be adjudicated by an independent Clinical Events Committee.

Funding and sponsor

RAPID N-STEMI is funded by the British Heart Foundation (grant number: CS/17/1/32445). The study sponsor is University Hospitals of Leicester National Health Service (NHS) Trust. The study is registered at ClinicalTrials.gov (NCT03707314). All procedures have been reviewed and approved by the UK National Research Ethics Service committee East of England (18/EE/0222).

Trial progress and impact of the COVID-19 pandemic

In March 2020, non-COVID-19 clinical research in the UK was suspended as NHS staff and resources were repurposed to frontline services in preparation for the volume of COVID-19 patients expected to place severe pressure on the NHS.[35] During this time, admissions with N-STEMI declined substantially.[36 37] Following the first wave in the UK, RAPID N-STEMI restarted in late July 2020 at a limited number of sites that had sufficient resource to recommence recruitment. However, due to the impending second wave of the COVID-19 pandemic the trial was once again suspended in December 2020. Discussions with the funding body took place regarding the strategy for a successful restart, with it agreed an interim pooled event rate (blinded to group allocation) should be calculated. Lower than anticipated event rates were documented and it was agreed with the funder that enrolment should be discontinued for two reasons: 1) the effect of the pandemic on clinical services and 2) the rate of the primary outcome. In summary, 425 (18.4% intended) patients were enrolled to the main trial, with 61 of these participants included in the CMR substudy. The intention is

to perform in depth analyses of the available data from these populations and present these in the near future.

Discussion

The optimal timing of revascularisation in higher-risk N-STEMI is a controversial topic, not least because international guidelines that mandate an early (<24 hr) invasive strategy are not supported by prospective randomised-controlled clinical trial data that specifically investigate this population.[2 3] RAPID N-STEMI addresses this knowledge gap. However, like many other clinical trials during the COVID-19 pandemic, RAPID N-STEMI was discontinued due to the emergency restructuring of healthcare and clinical research services. Despite falling short of the recruitment target, RAPID N-STEMI has randomised 425 GRACE score defined higher-risk patients admitted with N-STEMI, making it the third largest study to specifically investigate this important patient population. It will therefore provide a significant contribution to the current evidence base, with dissemination of results planned for 2022.

COVID-19: implications for cardiovascular research

The major challenges faced due to COVID-19 were three-fold. First, significant reductions in admissions with acute coronary syndrome and HF occurred during the pandemic in the UK, with decreases of over to 40% in both disease entities, presumably due to fear of contagion in healthcare settings.[36 38 39] Not only did this reduce potential research participants, but such declines in admissions become a critical issue for clinical trial event reporting and thus may be a contributory factor to the lower event rates observed in RAPID N-STEMI.

Second, the NHS underwent the largest workforce redeployment since its inception to support severely pressurised frontline services treat the vast numbers of COVID-19 patients attending UK hospitals. Research staff were moved to such clinical areas, resulting in all non-COVID-19 related research being left severely disrupted and placed on hiatus until further notice,[40] the ramifications of which are sure to be felt long after the initial effects of the COVID-19 pandemic have abated.[41]

Third, and perhaps most fundamentally for RAPID N-STEMI, elective cardiology activity across the UK was effectively cancelled from the beginning of the first wave in March 2020.[35] Suspension of planned cases created greater catheter laboratory capacity for acute MI patients and dramatically reduced the standard of care timing to ICA for N-STEMI in the UK.[42] Such changes to catheter laboratory throughput and working patterns resulted in an unmanageable task of ensuring adequate time separation between the very early ICA and standard of care ICA arms in RAPID N-STEMI – essentially the control arm was accelerated. Since any potential differences in clinical outcomes are related to the difference in timing between the trial arms, new systems of care enforced by the COVID-19 pandemic left the RAPID N-STEMI investigators in a position whereby restarting recruitment would inevitably jeopardise the scientific validity of the trial.

Very early revascularisation in higher-risk N-STEMI: will optimal timing ever be defined?

RAPID N-STEMI is the fifth randomised trial to investigate the timing of an invasive strategy in GRACE score defined higher-risk N-STEMI patients, albeit the first to specifically investigate this higher-risk population. Prior studies report pre-specified GRACE >140 subgroup analyses (**Table 1**). Favourable results were observed in TIMACS and VERDICT,[9 15] while ELISA-3 and

RIDDLE N-STEMI showed signals of benefit although were limited by small sample sizes.[12 14] Since clinical event rates are greater in high-risk N-STEMI, it may be expected that an adequately powered study to detect a clinically meaningful difference is achievable. Yet, in recent years outcomes following N-STEMI have improved, largely due to enhanced therapeutics and interventional techniques.[43] Therefore, demonstration of superiority for hard clinical endpoints from a very early invasive strategy may not be feasible in the contemporary era because the logistics of delivering an appropriately powered trial may be prohibitive.

The choice of trial endpoints is also of note. The EARLY trial indicated benefit from a very early invasive approach in European Society of Cardiology defined high-risk patients (median GRACE score 122), but such benefit was driven by the softer endpoint of recurrent ischaemic events in a cohort that did not receive P2Y12 inhibitor pre-treatment.[17] One may question the clinical relevance of an endpoint, and as such it was not included in the composite primary endpoint of RAPID N-STEMI. Given that practice in many centres is now shifting to a strategy of early ICA in higher-risk N-STEMI patient groups, and that this strategy is now widely accepted as without excess risk, it appears unlikely that the optimal timing of revascularisation in higher-risk N-STEMI will ever be robustly defined.

Conclusion

RAPID N-STEMI and its mechanistic CMR substudy will provide further insights into GRACE score defined higher-risk N-STEMI and provide an additional contribution to the evidence base.

References

1. Fox KAA, Clayton TC, Damman P, et al. Long-term outcome of a routine versus selective invasive strategy in patients with non-ST-segment elevation acute coronary syndrome a meta-analysis of individual patient data. *J Am Coll Cardiol* 2010;55(22):2435-45. doi: 10.1016/j.jacc.2010.03.007
2. Collet JP, Thiele H, Barbato E, et al. 2020 ESC Guidelines for the management of acute coronary syndromes in patients presenting without persistent ST-segment elevation. *Eur Heart J* 2020 doi: 10.1093/eurheartj/ehaa575 [published Online First: 2020/08/30]
3. Amsterdam EA, Wenger NK, Brindis RG, et al. 2014 AHA/ACC Guideline for the Management of Patients with Non-ST-Elevation Acute Coronary Syndromes: a report of the American College of Cardiology/American Heart Association Task Force on Practice Guidelines. *J Am Coll Cardiol* 2014;64(24):e139-e228. doi: 10.1016/j.jacc.2014.09.017
4. Hall M, Bebb OJ, Dondo TB, et al. Guideline-indicated treatments and diagnostics, GRACE risk score, and survival for non-ST elevation myocardial infarction. *Eur Heart J* 2018;39(42):3798-806. doi: 10.1093/eurheartj/ehy517 [published Online First: 2018/09/12]
5. van 't Hof AW, de Vries ST, Dambrink JH, et al. A comparison of two invasive strategies in patients with non-ST elevation acute coronary syndromes: results of the Early or Late Intervention in unStable Angina (ELISA) pilot study. 2b/3a upstream therapy and acute coronary syndromes. *Eur Heart J* 2003;24(15):1401-5.
6. Neumann FJ, Kastrati A, Pogatsa-Murray G, et al. Evaluation of prolonged antithrombotic pretreatment ("cooling-off" strategy) before intervention in patients with unstable coronary syndromes: a randomized controlled trial. *JAMA* 2003;290(12):1593-9. doi: 10.1001/jama.290.12.1593
7. Montalescot G, Cayla G, Collet JP, et al. Immediate vs delayed intervention for acute coronary syndromes: a randomized clinical trial. *JAMA* 2009;302(9):947-54. doi: 10.1001/jama.2009.1267
8. Riezebos RK, Ronner E, Ter Bals E, et al. Immediate versus deferred coronary angioplasty in non-ST-segment elevation acute coronary syndromes. *Heart* 2009;95(10):807-12. doi: 10.1136/hrt.2008.154815
9. Mehta SR, Granger CB, Boden WE, et al. Early versus delayed invasive intervention in acute coronary syndromes. *N Engl J Med* 2009;360(21):2165-75. doi: 10.1056/NEJMoa0807986
10. Sciahbasi A, Madonna M, De Vita M, et al. Comparison of immediate vs early invasive strategy in patients with first acute non-ST-elevation myocardial infarction. *Clin Cardiol* 2010;33(10):650-5. doi: 10.1002/clc.20785 [published Online First: 2010/10/21]
11. Thiele H, Rach J, Klein N, et al. Optimal timing of invasive angiography in stable non-ST-elevation myocardial infarction: the Leipzig Immediate versus early and late PercutaneouS coronary Intervention triAl in NSTEMI (LIPSIA-NSTEMI Trial). *Eur Heart J* 2012;33(16):2035-43. doi: 10.1093/eurheartj/ehr418
12. Badings EA, The SH, Dambrink JH, et al. Early or late intervention in high-risk non-ST-elevation acute coronary syndromes: results of the ELISA-3 trial. *EuroIntervention* 2013;9(1):54-61. doi: 10.4244/EIJV9I1A9

13. Reuter PG, Rouchy C, Cattan S, et al. Early invasive strategy in high-risk acute coronary syndrome without ST-segment elevation. The Sisca randomized trial. *Int J Cardiol* 2015;182:414-8. doi: 10.1016/j.ijcard.2014.12.089
14. Milosevic A, Vasiljevic-Pokrajcic Z, Milasinovic D, et al. Immediate Versus Delayed Invasive Intervention for Non-STEMI Patients: The RIDDLE-NSTEMI Study. *JACC Cardiovasc Interv* 2016;9(6):541-9. doi: 10.1016/j.jcin.2015.11.018
15. Kofoed KF, Kelbaek H, Hansen PR, et al. Early Versus Standard Care Invasive Examination and Treatment of Patients With Non-ST-Segment Elevation Acute Coronary Syndrome. *Circulation* 2018;138(24):2741-50. doi: 10.1161/CIRCULATIONAHA.118.037152
16. Rasmussen MB, Stengaard C, Sorensen JT, et al. Comparison of Acute Versus Subacute Coronary Angiography in Patients With NON-ST-Elevation Myocardial Infarction (from the NONSTEMI Trial). *Am J Cardiol* 2019;124(6):825-32. doi: 10.1016/j.amjcard.2019.06.007 [published Online First: 2019/07/22]
17. Lemesle G, Laine M, Pankert M, et al. Optimal Timing of Intervention in NSTE-ACS Without Pre-Treatment: The EARLY Randomized Trial. *JACC Cardiovasc Interv* 2020;13(8):907-17. doi: 10.1016/j.jcin.2020.01.231 [published Online First: 2020/04/25]
18. Fagel ND, Amoroso G, Vink MA, et al. An immediate or early invasive strategy in non-ST-elevation acute coronary syndrome: The OPTIMA-2 randomized controlled trial. *Am Heart J* 2021;234:42-50.
19. Jobs A, Mehta SR, Montalescot G, et al. Optimal timing of an invasive strategy in patients with non-ST-elevation acute coronary syndrome: a meta-analysis of randomised trials. *Lancet* 2017;390(10096):737-46. doi: 10.1016/S0140-6736(17)31490-3
20. Neumann FJ, Sousa-Uva M, Ahlsson A, et al. 2018 ESC/EACTS Guidelines on myocardial revascularization. *Eur Heart J* 2019;40(2):87-165. doi: 10.1093/eurheartj/ehy394 [published Online First: 2018/08/31]
21. Herdman M, Gudex C, Lloyd A, et al. Development and preliminary testing of the new five-level version of EQ-5D (EQ-5D-5L). *Qual Life Res* 2011;20(10):1727-36. doi: 10.1007/s11136-011-9903-x [published Online First: 2011/04/12]
22. Spertus JA, Winder JA, Dewhurst TA, et al. Development and evaluation of the Seattle Angina Questionnaire: a new functional status measure for coronary artery disease. *J Am Coll Cardiol* 1995;25(2):333-41. doi: 10.1016/0735-1097(94)00397-9 [published Online First: 1995/02/01]
23. Fox KA, Fitzgerald G, Puymirat E, et al. Should patients with acute coronary disease be stratified for management according to their risk? Derivation, external validation and outcomes using the updated GRACE risk score. *BMJ Open* 2014;4(2):e004425. doi: 10.1136/bmjopen-2013-004425
24. Stone PH, Raabe DS, Jaffe AS, et al. Prognostic significance of location and type of myocardial infarction: independent adverse outcome associated with anterior location. *J Am Coll Cardiol* 1988;11(3):453-63.
25. Mak KH, Topol EJ. Emerging concepts in the management of acute myocardial infarction in patients with diabetes mellitus. *J Am Coll Cardiol* 2000;35(3):563-8.
26. Bagai A, Huang Z, Lokhnygina Y, et al. Magnitude of troponin elevation and long-term clinical outcomes in acute coronary syndrome patients treated with and without revascularization. *Circ Cardiovasc Interv* 2015;8(6):e002314. doi: 10.1161/CIRCINTERVENTIONS.115.002314

27. Kaul P, Ezekowitz JA, Armstrong PW, et al. Incidence of heart failure and mortality after acute coronary syndromes. *Am Heart J* 2013;165(3):385.e2. doi: 10.1016/j.ahj.2012.12.005
28. Plein S, Younger JF, Sparrow P, et al. Cardiovascular magnetic resonance of scar and ischemia burden early after acute ST elevation and non-ST elevation myocardial infarction. *J Cardiovasc Magn Reson* 2008;10:47. doi: 10.1186/1532-429X-10-47
29. Eitel I, Stiermaier T, Lange T, et al. Cardiac Magnetic Resonance Myocardial Feature Tracking for Optimized Prediction of Cardiovascular Events Following Myocardial Infarction. *JACC Cardiovasc Imaging* 2018;11(10):1433-44. doi: 10.1016/j.jcmg.2017.11.034 [published Online First: 2018/02/20]
30. Eitel I, Desch S, Fuernau G, et al. Prognostic significance and determinants of myocardial salvage assessed by cardiovascular magnetic resonance in acute reperfused myocardial infarction. *J Am Coll Cardiol* 2010;55(22):2470-9. doi: 10.1016/j.jacc.2010.01.049
31. Mather AN, Fairbairn TA, Artis NJ, et al. Timing of Cardiovascular MR Imaging after Acute Myocardial Infarction : Effect on Estimates of Infarct Characteristics and Prediction of Late Ventricular Remodeling. *Radiology* 2011;261:116-26.
32. Selvanayagam JB, Porto I, Channon K, et al. Troponin elevation after percutaneous coronary intervention directly represents the extent of irreversible myocardial injury: insights from cardiovascular magnetic resonance imaging. *Circulation* 2005;111(8):1027-32. doi: 10.1161/01.CIR.0000156328.28485.AD [published Online First: 2005/02/23]
33. McCann GP, Khan JN, Greenwood JP, et al. Complete Versus Lesion-Only Primary PCI: The Randomized Cardiovascular MR CvLPRIT Substudy. *J Am Coll Cardiol* 2015;66(24):2713-24. doi: 10.1016/j.jacc.2015.09.099 [published Online First: 2015/12/25]
34. Singh A, Greenwood JP, Berry C, et al. Comparison of exercise testing and CMR measured myocardial perfusion reserve for predicting outcome in asymptomatic aortic stenosis: the PRognostic Importance of MIcrovascular Dysfunction in Aortic Stenosis (PRIMID AS) Study. *Eur Heart J* 2017;38(16):1222-29. doi: 10.1093/eurheartj/ehx001 [published Online First: 2017/02/17]
35. NHS England. Next steps on NHS response to COVID-19: Letter from Sir Simon Stevens and Amanda Pritchard 2020 [Available from: https://www.england.nhs.uk/coronavirus/wp-content/uploads/sites/52/2020/03/20200317-NHS-COVID-letter-FINAL.pdf accessed 8th December 2020.
36. Mafham MM, Spata E, Goldacre R, et al. COVID-19 pandemic and admission rates for and management of acute coronary syndromes in England. *Lancet* 2020 doi: 10.1016/S0140-6736(20)31356-8 [published Online First: 2020/07/18]
37. Wu J, Mamas M, Rashid M, et al. Patient response, treatments and mortality for acute myocardial infarction during the COVID-19 pandemic. *Eur Heart J Qual Care Clin Outcomes* 2020 doi: 10.1093/ehjqcco/qcaa062 [published Online First: 2020/07/31]
38. Shoaib A, Van Spall HGC, Wu J, et al. Substantial decline in hospital admissions for heart failure accompanied by increased community mortality during COVID-19 pandemic. *Eur Heart J Qual Care Clin Outcomes* 2021 doi: 10.1093/ehjqcco/qcab040 [published Online First: 2021/05/28]

39. Wu J, Mamas MA, de Belder MA, et al. Second Decline in Admissions With Heart Failure and Myocardial Infarction During the COVID-19 Pandemic. *J Am Coll Cardiol* 2021;77(8):1141-43. doi: 10.1016/j.jacc.2020.12.039 [published Online First: 2021/01/24]

40. National Institute for Health Research. DHSC issues guidance on the impact of COVID-19 on research funded or supported by NIHR, updated 19 Mar 2020 [Available from: https://www.nihr.ac.uk/news/dhsc-issues-guidance-on-the-impact-on-covid-19-on-research-funded-or-supported-by-nihr/24469.

41. van Dorn A. COVID-19 and readjusting clinical trials. *Lancet* 2020;396(10250):523-24. doi: 10.1016/S0140-6736(20)31787-6 [published Online First: 2020/08/24]

42. Kwok CS, Gale CP, Curzen N, et al. Impact of the COVID-19 Pandemic on Percutaneous Coronary Intervention in England: Insights From the British Cardiovascular Intervention Society PCI Database Cohort. *Circ Cardiovasc Interv* 2020;13(11):e009654. doi: 10.1161/CIRCINTERVENTIONS.120.009654 [published Online First: 2020/11/04]

43. Hall M, Dondo TB, Yan AT, et al. Association of Clinical Factors and Therapeutic Strategies With Improvements in Survival Following Non-ST-Elevation Myocardial Infarction, 2003-2013. *JAMA* 2016;316(10):1073-82. doi: 10.1001/jama.2016.10766 [published Online First: 2016/08/31]

Acknowledgements

The authors would like to acknowledge the support of the National Institute for Health Research Biomedical Research Centre in Leicester and the Leeds National Institute for Health Research Clinical Research Facility.

Author statement

AHG, GPM, ASB, APB, NC, CB, MF and MD conceived the idea for the study. AHG, GPM, ASB and MF designed the study protocol. TAK drafted the manuscript. SB provided statistical support. All authors critically reviewed and approved the final draft of the manuscript.

Funding

The work was supported by the British Heart Foundation (grant number: CS/17/1/32445). The grant providers were not involved in study design, data acquisition or management, or analysis and writing of final reports.

Competing interests

CB is employed by the University of Glasgow which holds consultancy and research agreements for his work with Abbott Vascular, AstraZeneca, Boehringer Ingelheim, Causeway Therapeutics, Coroventis, Genentech, GSK, HeartFlow, Menarini, Neovasc, Siemens Healthcare, and Valo Health. The other co-authors have no relevant disclosures.

Ethics and dissemination

The trial is conducted in accordance with the principles of the 1996 Helsinki Declarations, International Conference on Harmonisation-Good Clinical Practice Guidelines (ICH-GCP). It is

anticipated that data collection will be completed by December 2021. The study results will be submitted for publication within 6 months of completion.

Data statement

The deidentified participant data will be available upon reasonable request.

Figure Legends

Figure 1: RAPID N-STEMI study flow diagram

CMR: cardiac magnetic resonance; ECG: electrocardiogram; hs-Tn: high sensitivity troponin N-STEMI: non-ST elevation myocardial infarction; OMT: optimal medical therapy; SAQ: Seattle Angina Questionnaire; STEMI: ST elevation myocardial infarction; ULN: upper limit of normal

Table 1: Higher-risk N-STEMI (GRACE score >140) subgroup analyses from randomised studies comparing early and delayed invasive strategies

Trial	Patients	Time to ICA: early (median & IQR, h)	Time to ICA: delayed (median & IQR, h)	Primary outcome	Results
TIMACS 2009[9]	961	14.0 (3.0 – 21.0)	50.0 (41.0 – 81.0)	6-month death, non-fatal MI, stroke	Early = 13.9%<br>Delayed = 21.0%<br>HR 0.65, 95% CI 0.48-0.89, p = 0.006
ELISA-3 2013[12]	224	2.6 (1.2 – 6.2)	54.9 (44.2 – 74.5)	30-day death, non-fatal MI, recurrent ischaemia	Early = 10.5%<br>Delayed = 19.1%<br>HR 0.55, 95% CI 0.29-1.10, p=0.26
RIDDLE-NSTEMI 2016[14]	123	1.4 (1.0 – 2.2)	61.0 (35.8 – 85.0)	30-day death, non-fatal MI	Early = 10.7%<br>Delayed = 17.9%<br>HR 0.56, 95% CI 0.21-1.51 p=0.12
VERDICT 2018[15]	1025	4.7 (3.0 – 12.2)	61.6 (39.4 – 87.8)	Death, non-fatal MI, refractory ischaemia, admission for heart failure at median 4.3 years	Early = 34.0%<br>Delayed = 40.1%<br>HR 0.81, 95% CI 0.66-0.99, p = 0.023

CI: confidence interval; HR: hazard ratio; ICA: invasive coronary angiography; IQR: interquartile range; MI: myocardial infarction

Table 2: RAPID N-STEMI inclusion and exclusion criteria

Inclusion criteria	Exclusion criteria
>18 years of age	ST elevation myocardial infarction
Clinical diagnosis of N-STEMI comprising:<br>▪ Cardiovascular symptoms suggestive of myocardial ischaemia<br>▪ Elevated high-sensitivity troponin I or T	Evident type 2 myocardial infarction
Symptoms <12 hours prior to admission	Previous known cardiomyopathy
GRACE 2.0 score ≥118 or if GRACE 2.0 score ≥90 but <118 must have at least one high-risk feature:<br>▪ Anterior location of ECG changes (V2-V5)<br>▪ ST segment depression in 2 contiguous leads of 0.15mV/1.5mm<br>▪ Diabetes mellitus on medication<br>▪ Elevated high-sensitivity troponin 3x upper limit of normal	Need for urgent PCI according to ESC Guidelines (haemodynamic instability, VT, VF, recurrent or persistent pain)
Intention to perform angiography and, if indicated, follow-on revascularisation	Cardiogenic shock
Provision of verbal assent followed by written informed consent	Severe valvular heart disease
	Any contraindication to PCI
	Current participation in another intervention trial

ECG: electrocardiogram; ESC: European Society of Cardiology; GRACE: Global Registry of Acute Coronary Events; N-STEMI: non-ST elevation myocardial infarction; PCI: percutaneous coronary intervention; VF: ventricular fibrillation; VT: ventricular tachycardia

Table 3: RAPID N-STEMI study endpoints

Primary outcome	Secondary outcomes
All-cause mortality, non-fatal myocardial infarction, and admission for heart failure at 12 months	Individual components of primary composite outcome
	Cardiovascular mortality
	Ischaemia-driven revascularisation
	BARC 3-5 major bleeding
	Stroke
	Length of inpatient stay
	Admission for any cause
	Events prior to angiography
	Quality of life (Seattle Angina Questionnaire and EQ-5D-5L questionnaires)
	Cost-efficacy analysis
	Proportion of patients requiring emergency revascularisation in group B
	Total VARC-2 classified access site complications
	Major VARC-2 classified access site complications

BARC: Bleeding Academic Research Consortium; VARC: Vascular Access Research Consortium

Table 4: RAPID N-STEMI CMR study endpoints

**Primary outcome**	**Secondary outcomes**
Infarct size (% left ventricular mass)	Left ventricular volumes and ejection fraction
	Myocardial salvage index
	Extracellular volume
	Ischaemic burden
	Global myocardial strain

For peer review only - http://bmjopen.bmj.com/site/about/guidelines.xhtml

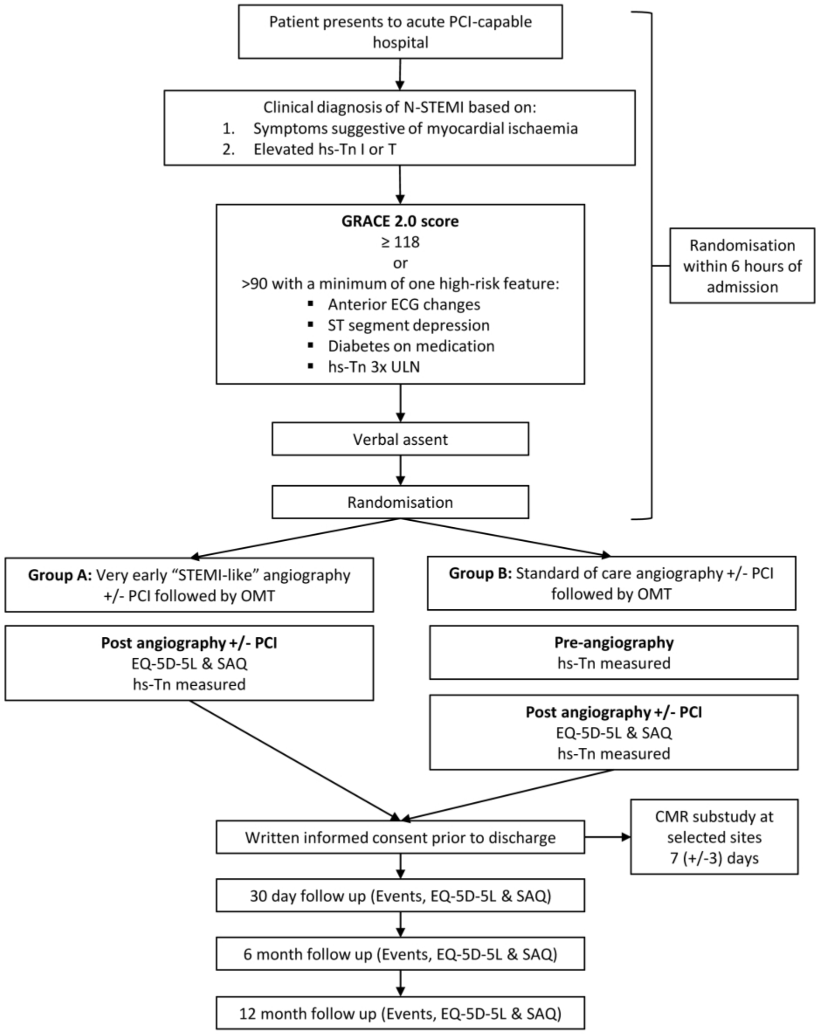

190x268mm (300 x 300 DPI)

Reporting checklist for protocol of a clinical trial.

Based on the SPIRIT guidelines.

Instructions to authors

Complete this checklist by entering the page numbers from your manuscript where readers will find each of the items listed below.

Your article may not currently address all the items on the checklist. Please modify your text to include the missing information. If you are certain that an item does not apply, please write "n/a" and provide a short explanation.

Upload your completed checklist as an extra file when you submit to a journal.

In your methods section, say that you used the SPIRITreporting guidelines, and cite them as:

Chan A-W, Tetzlaff JM, Gøtzsche PC, Altman DG, Mann H, Berlin J, Dickersin K, Hróbjartsson A, Schulz KF, Parulekar WR, Krleža-Jerić K, Laupacis A, Moher D. SPIRIT 2013 Explanation and Elaboration: Guidance for protocols of clinical trials. BMJ. 2013;346:e7586

		Reporting Item	Page Number
**Administrative information**			
Title	#1	Descriptive title identifying the study design, population, interventions, and, if applicable, trial acronym	1
Trial registration	#2a	Trial identifier and registry name. If not yet registered, name of intended registry	2
Trial registration: data set	#2b	All items from the World Health Organization Trial Registration Data Set	2
Protocol version	#3	Date and version identifier	3
Funding	#4	Sources and types of financial, material, and other support	20
Roles and responsibilities: contributorship	#5a	Names, affiliations, and roles of protocol contributors	20

For peer review only

Roles and responsibilities: sponsor contact information	#5b	Name and contact information for the trial sponsor	8
Roles and responsibilities: sponsor and funder	#5c	Role of study sponsor and funders, if any, in study design; collection, management, analysis, and interpretation of data; writing of the report; and the decision to submit the report for publication, including whether they will have ultimate authority over any of these activities	20
Roles and responsibilities: committees	#5d	Composition, roles, and responsibilities of the coordinating centre, steering committee, endpoint adjudication committee, data management team, and other individuals or groups overseeing the trial, if applicable (see Item 21a for data monitoring committee)	11
**Introduction**			
Background and rationale	#6a	Description of research question and justification for undertaking the trial, including summary of relevant studies (published and unpublished) examining benefits and harms for each intervention	5
Background and rationale: choice of comparators	#6b	Explanation for choice of comparators	5
Objectives	#7	Specific objectives or hypotheses	6
Trial design	#8	Description of trial design including type of trial (eg, parallel group, crossover, factorial, single group), allocation ratio, and framework (eg, superiority, equivalence, non-inferiority, exploratory)	6
**Methods: Participants, interventions, and outcomes**			
Study setting	#9	Description of study settings (eg, community clinic, academic hospital) and list of countries where data will be collected. Reference to where list of study sites can be obtained	6
Eligibility criteria	#10	Inclusion and exclusion criteria for participants. If applicable, eligibility criteria for study centres and individuals who will	7

For peer review only

		perform the interventions (eg, surgeons, psychotherapists)	
Interventions: description	#11a	Interventions for each group with sufficient detail to allow replication, including how and when they will be administered	7
Interventions: modifications	#11b	Criteria for discontinuing or modifying allocated interventions for a given trial participant (eg, drug dose change in response to harms, participant request, or improving / worsening disease)	7
Interventions: adherance	#11c	Strategies to improve adherence to intervention protocols, and any procedures for monitoring adherence (eg, drug tablet return; laboratory tests)	7
Interventions: concomitant care	#11d	Relevant concomitant care and interventions that are permitted or prohibited during the trial	7
Outcomes	#12	Primary, secondary, and other outcomes, including the specific measurement variable (eg, systolic blood pressure), analysis metric (eg, change from baseline, final value, time to event), method of aggregation (eg, median, proportion), and time point for each outcome. Explanation of the clinical relevance of chosen efficacy and harm outcomes is strongly recommended	10
Participant timeline	#13	Time schedule of enrolment, interventions (including any run-ins and washouts), assessments, and visits for participants. A schematic diagram is highly recommended (see Figure)	10
Sample size	#14	Estimated number of participants needed to achieve study objectives and how it was determined, including clinical and statistical assumptions supporting any sample size calculations	10
Recruitment	#15	Strategies for achieving adequate participant enrolment to reach target sample size	10
**Methods: Assignment of interventions (for controlled trials)**			
Allocation: sequence generation	#16a	Method of generating the allocation sequence (eg, computer-generated random numbers), and list of any factors for stratification. To reduce predictability of a random sequence, details of any planned restriction (eg, blocking) should be provided in a separate document that is unavailable to those who enrol participants or assign interventions	7

For peer review only

Allocation concealment mechanism	#16b	Mechanism of implementing the allocation sequence (eg, central telephone; sequentially numbered, opaque, sealed envelopes), describing any steps to conceal the sequence until interventions are assigned	7
Allocation: implementation	#16c	Who will generate the allocation sequence, who will enrol participants, and who will assign participants to interventions	7
Blinding (masking)	#17a	Who will be blinded after assignment to interventions (eg, trial participants, care providers, outcome assessors, data analysts), and how	7
Blinding (masking): emergency unblinding	#17b	If blinded, circumstances under which unblinding is permissible, and procedure for revealing a participant's allocated intervention during the trial	7
**Methods: Data collection, management, and analysis**			
Data collection plan	#18a	Plans for assessment and collection of outcome, baseline, and other trial data, including any related processes to promote data quality (eg, duplicate measurements, training of assessors) and a description of study instruments (eg, questionnaires, laboratory tests) along with their reliability and validity, if known. Reference to where data collection forms can be found, if not in the protocol	8
Data collection plan: retention	#18b	Plans to promote participant retention and complete follow-up, including list of any outcome data to be collected for participants who discontinue or deviate from intervention protocols	8
Data management	#19	Plans for data entry, coding, security, and storage, including any related processes to promote data quality (eg, double data entry; range checks for data values). Reference to where details of data management procedures can be found, if not in the protocol	8
Statistics: outcomes	#20a	Statistical methods for analysing primary and secondary outcomes. Reference to where other details of the statistical analysis plan can be found, if not in the protocol	11
Statistics: additional analyses	#20b	Methods for any additional analyses (eg, subgroup and adjusted analyses)	11

For peer review only

Statistics: analysis population and missing data	#20c	Definition of analysis population relating to protocol non-adherence (eg, as randomised analysis), and any statistical methods to handle missing data (eg, multiple imputation)	11
**Methods: Monitoring**			
Data monitoring: formal committee	#21a	Composition of data monitoring committee (DMC); summary of its role and reporting structure; statement of whether it is independent from the sponsor and competing interests; and reference to where further details about its charter can be found, if not in the protocol. Alternatively, an explanation of why a DMC is not needed	11
Data monitoring: interim analysis	#21b	Description of any interim analyses and stopping guidelines, including who will have access to these interim results and make the final decision to terminate the trial	n/a
Harms	#22	Plans for collecting, assessing, reporting, and managing solicited and spontaneously reported adverse events and other unintended effects of trial interventions or trial conduct	n/a
Auditing	#23	Frequency and procedures for auditing trial conduct, if any, and whether the process will be independent from investigators and the sponsor	n/a
**Ethics and dissemination**			
Research ethics approval	#24	Plans for seeking research ethics committee / institutional review board (REC / IRB) approval	8
Protocol amendments	#25	Plans for communicating important protocol modifications (eg, changes to eligibility criteria, outcomes, analyses) to relevant parties (eg, investigators, REC / IRBs, trial participants, trial registries, journals, regulators)	n/a
Consent or assent	#26a	Who will obtain informed consent or assent from potential trial participants or authorised surrogates, and how (see Item 32)	n/a
Consent or assent: ancillary studies	#26b	Additional consent provisions for collection and use of participant data and biological specimens in ancillary studies, if applicable	n/a
Confidentiality	#27	How personal information about potential and enrolled participants will be collected, shared, and maintained in order to protect confidentiality before, during, and after the trial	n/a

For peer review only

Declaration of interests	[#28](#28)	Financial and other competing interests for principal investigators for the overall trial and each study site	20
Data access	[#29](#29)	Statement of who will have access to the final trial dataset, and disclosure of contractual agreements that limit such access for investigators	21
Ancillary and post trial care	[#30](#30)	Provisions, if any, for ancillary and post-trial care, and for compensation to those who suffer harm from trial participation	n/a
Dissemination policy: trial results	[#31a](#31a)	Plans for investigators and sponsor to communicate trial results to participants, healthcare professionals, the public, and other relevant groups (eg, via publication, reporting in results databases, or other data sharing arrangements), including any publication restrictions	20
Dissemination policy: authorship	[#31b](#31b)	Authorship eligibility guidelines and any intended use of professional writers	n/a
Dissemination policy: reproducible research	[#31c](#31c)	Plans, if any, for granting public access to the full protocol, participant-level dataset, and statistical code	20
**Appendices**			
Informed consent materials	[#32](#32)	Model consent form and other related documentation given to participants and authorised surrogates	n/a
Biological specimens	[#33](#33)	Plans for collection, laboratory evaluation, and storage of biological specimens for genetic or molecular analysis in the current trial and for future use in ancillary studies, if applicable	n/a

The SPIRIT Explanation and Elaboration paper is distributed under the terms of the Creative Commons Attribution License CC-BY-NC. This checklist was completed on 26. July 2021 using https://www.goodreports.org/, a tool made by the EQUATOR Network in collaboration with Penelope.ai

For peer review only

Very early invasive angiography versus standard of care in higher-risk non-ST elevation myocardial infarction: study protocol for the prospective multicentre randomised controlled RAPID N-STEMI trial

Journal:	*BMJ Open*
Manuscript ID	bmjopen-2021-055878.R1
Article Type:	Protocol
Date Submitted by the Author:	05-Jan-2022
Complete List of Authors:	Kite, Thomas; Glenfield Hospital, Department of Cardiovascular Sciences<br>Banning, Amerjeet S.; Glenfield Hospital, Department of Cardiovascular Sciences<br>Ladwiniec, Andrew; Glenfield Hospital, Department of Cardiovascular Sciences<br>Gale, Chris; University of Leeds<br>Greenwood, John; Leeds Teaching Hospitals NHS Trust, Cardiology; University of Leeds, Biomedical Imaging Sciences<br>Dalby, Miles; Royal Brompton & Harefield NHS Foundation Trust, Imperial College London; Royal Brompton & Harefield NHS Foundation Trust<br>Hobson, Rachel; University of Leicester<br>Barber, Shaun; University of Leicester<br>Parker, Emma; Glenfield Hospital, Department of Cardiovascular Sciences<br>Berry, Colin; University of Glasgow, British Heart Foundation Glasgow Cardiovascular Research Centre; University of Glasgow<br>Flather, Marcus; University of East Anglia, Norwich Medical School<br>Curzen, Nick; University Hospital Southampton NHS F Trust, Wessex Cardiac Unit<br>Banning, AP; Oxford University Hospitals NHS Foundation Trust, Cardiology Department, Oxford Heart Centre<br>McCann, Gerry; Glenfield Hospital, Department of Cardiovascular Sciences<br>GERSHLICK, ANTHONY; University Hospitals of Leicester, Cardiology
**Primary Subject Heading**:	Cardiovascular medicine
Secondary Subject Heading:	Evidence based practice
Keywords:	Ischaemic heart disease < CARDIOLOGY, Myocardial infarction < CARDIOLOGY, Coronary intervention < CARDIOLOGY, Coronary heart disease < CARDIOLOGY

Very early invasive angiography versus standard of care in higher-risk non-ST elevation myocardial infarction: study protocol for the prospective multicentre randomised controlled RAPID N-STEMI trial

Thomas A. Kite BMedSci (Hons) BM BS MRCP[1], Amerjeet S. Banning MBBS PhD MRCP[1], Andrew Ladwiniec MA MD FRCP[1], Chris P. Gale BSc PhD FRCP[2], John P. Greenwood MBChB PhD FRCP[2], Miles Dalby BSc MBBS MD[3], Rachel Hobson BA(Hons) [4], Shaun Barber BSc MSc PhD[4], Emma Parker[1], Colin Berry MBChB PhD[5], Marcus Flather MBBS MBA FRCP[6], Nick Curzen BM (Hons) PhD[7], Adrian P. Banning MB BS, MD, FRCP[8], Gerry P. McCann MB ChB, MD[1], Anthony H. Gershlick BSc MB BS FRCP[1]

Affiliations:

1. Department of Cardiovascular Sciences, University of Leicester and the Leicester Biomedical Research Centre, Glenfield Hospital, Leicester, UK
2. Leeds Institute of Cardiovascular and Metabolic Medicine, University of Leeds and the Department of Cardiology Leeds Teaching Hospitals NHS Trust, Leeds, UK
3. Royal Brompton and Harefield NHS Foundation Trust, London, UK
4. Leicester Clinical Trials Unit, University of Leicester, University Road, Leicester, UK
5. Institute of Cardiovascular and Medical Sciences, University of Glasgow, Glasgow, UK
6. Norwich Medical School, University of East Anglia, Norwich, UK
7. Faculty of Medicine, University of Southampton and University Hospital Southampton NHS Trust Southampton, UK
8. John Radcliffe Hospital, Oxford University Hospitals NHS Trust, Oxford, UK

Corresponding author:
Dr Thomas A. Kite
Department of Cardiovascular Sciences, University of Leicester and the Leicester Biomedical Research Centre, Glenfield Hospital, Leicester, LE3 9QP, UK
Email: tom.kite@nhs.net

For peer review only

Abstract

Background: There are a paucity of randomised data on the optimal timing of invasive coronary angiography (ICA) in higher-risk patients with non-ST elevation myocardial infarction (N-STEMI). International guideline recommendations for early ICA are primarily based on retrospective subgroup analyses of neutral trials.

Aims: The RAPID N-STEMI trial aims to determine whether very early percutaneous revascularisation improves clinical outcomes as compared to a standard of care strategy in higher-risk N-STEMI patients.

Methods and analysis: RAPID N-STEMI is a prospective, multi-centre, open-label, randomised-controlled, pragmatic strategy trial. Higher-risk N-STEMI patients, as defined by Global Registry of Acute Coronary Events (GRACE) 2.0 score ≥118, or >90 with at least one additional high-risk feature, were randomised to either: very early ICA +/- revascularisation or standard of care timing of ICA +/- revascularisation. The primary outcome is the proportion of participants with at least one of the following events (all-cause mortality, non-fatal myocardial infarction, and hospital admission for heart failure) at 12 months. Key secondary outcomes include major bleeding and stroke. A hypothesis generating cardiac magnetic resonance (CMR) substudy will provide mechanistic data on infarct size, myocardial salvage, and residual ischaemia post percutaneous coronary intervention. On 7th April 2021, the sponsor discontinued enrolment due to the impact of the COVID-19 pandemic and lower than expected event rates. 425 patients were enrolled, and 61 patients underwent CMR.

Ethics and dissemination: The trial has been reviewed and approved by the East of England Cambridge East Research Ethics Committee (18/EE/0222). The study results will be submitted for publication within 6 months of completion.

Clinical trial registration
NCT03707314

Keywords
Non-ST elevation myocardial infarction; percutaneous coronary intervention; timing; invasive strategy; GRACE score

For peer review only

Strengths and limitations of this study:

-This randomised trial sought to test whether a very early invasive strategy in higher-risk N-STEMI patients improves clinical outcomes compared with standard care. An early invasive strategy in this group is recommended in international guidelines, but is as yet unsupported by the primary outcome of an appropriately sized randomised trial.

-Randomised-controlled pragmatic strategy design.

-A cardiac magnetic resonance sub-study will provide mechanistic data on infarct size, myocardial salvage, and residual ischaemia post-PCI.

-Due to the effects of the COVID-19 pandemic upon clinical services and a lower than expected primary outcome event rate, trial recruitment was terminated early after enrolment of 425 patients (18.4% of intended).

For peer review only

Background and rationale

Historical data indicate that an invasive strategy during index hospital admission in non-ST elevation myocardial infarction (N-STEMI) improves composite ischaemic outcomes, with maximal benefit seen in those at highest baseline risk for future major adverse cardiovascular events (MACE) (1). However, the optimal timing of invasive coronary angiography (ICA) and whether high-risk subgroups should be treated early remains controversial, despite it being a mandated management strategy in international guidelines (2, 3). Since N-STEMI is

characterised by a risk-treatment paradox whereby higher-risk patients are less likely to receive aggressive pharmacotherapy and invasive management (4), use and timing of ICA can differ significantly when compared to the precisely defined management of ST elevation myocardial infarction (STEMI). Clinically unstable patients require urgent revascularisation, whilst for others, the most appropriate timing of an invasive strategy is less certain.

A series of randomised trials have investigated this concept in unselected N-STEMI populations, thereby testing whether early revascularisation (<24 hours) improves clinical outcomes as compared to delayed or standard of care approaches (5-18). Differences in study design, inclusion criteria, timing of ICA and endpoint definitions have resulted in conflicting results that are challenging to interpret and apply to current practice. When these data are evaluated in totality, patient level meta-analysis has demonstrated no significant difference in death or myocardial infarction (MI) between the two strategies (19).

The application of the Global Registry of Acute Coronary Events (GRACE) score in prior trial subgroup analyses has potential for risk stratification of those patients that may benefit most from expeditious revascularisation (**Table 1**). A GRACE score >140 analysis of 961 patients from the TIMACS trial showed that an early invasive strategy (14 hours post randomisation) reduced the risk of death, non-fatal MI, and stroke at 6 months by 35% as compared to a delayed approach (9). Moreover, the VERDICT study produced a similar finding, albeit in a subgroup of 1025 GRACE >140 N-STEMI patients who underwent a very early invasive strategy (4.7 hours post randomisation) (15). However, such analyses should only be considered hypothesis generating since; 1) the primary outcome in both overall trial populations was

For peer review only

neutral, and 2) the studies were undertaken in the era of conventional troponin and CK-MB, with up to one-quarter of patients exhibiting no biomarker rise (9).

Given that currently available data are inconsistent and of insufficient scientific quality to inform best practice, a contemporary trial that prospectively investigates the timing of revascularisation in GRACE score defined high-risk N-STEMI is required to confirm or refute these prior observations.

Methods and analysis

Study design and inclusion criteria

The RAPID N-STEMI trial enrolled patients across 32 hospitals with on-site cardiac catheter laboratories in the United Kingdom (UK). Potential participants who experienced symptoms within 12 hours prior to admission were assessed on attendance to hospital and the research team alerted if a diagnosis of N-STEMI was suspected. N-STEMI was defined as: 1) the presence of cardiovascular symptoms suggestive of myocardial ischaemia and, 2) elevation in high-sensitivity troponin (hs-Tn) I or T. Risk stratification using the GRACE 2.0 score was then performed. Patients in whom the GRACE 2.0 score was ≥118, or ≥90 with at least one additional feature of high-risk presentation were deemed as higher risk. The full inclusion and exclusion criteria are listed in **Table 2.**

Patients were enrolled after obtaining verbal consent once eligibility was confirmed in the Emergency Department or appropriate receiving unit. Participants were then randomised in a 1:1 fashion to either: Group A) very early ICA with a view to revascularisation; or Group B) standard of care timing of ICA with a view to revascularisation. Research team members had

For peer review only

6 hours from hospital admission to randomise verbally consented patients who met all eligibility criteria (**Figure 1**).

Study procedures

Randomisation was performed via either a secure centralised web-based or telephone assisted system provided by http://www.sealedenvelope.com. Those assigned to very early angiography were transferred to the cardiac catheter laboratory as soon as possible. Teams were encouraged, but not mandated to achieve a randomisation to vascular sheath insertion time of less than 90 minutes. Timing of standard of care ICA was according to typical practice at individual UK centres but encouraged to be within 72 hours of admission. Percutaneous coronary intervention (PCI) and coronary artery bypass grafting (CABG) were performed according to current international guidelines (20). Requirement for multi-vessel revascularisation to a non-infarct related artery was at the individual operator's discretion. Optimal medical therapy, including the use of anti-platelet agents, was in accordance with current clinical guidelines (2). Drug eluting stents were used in all cases unless there were clear contraindications. As this was a pragmatic strategy trial, all procedures performed were part of guideline directed standard of care for N-STEMI.

Patients were invited to provide verbal consent to participate in the study after being read an abridged consent form prior to randomisation. This was to avoid delay in those participants that were subsequently allocated to a very early invasive strategy. In addition to the baseline hs-Tn required for study inclusion, samples were obtained post-angiography in both trial arms and prior to angiography in the standard of care arm. EQ-5D-5L (21) and Seattle Angina Questionnaires (22) to assess quality of life were completed after angiography in both arms.

For peer review only

All patients were then asked to provide written informed consent for continuation in the study prior to hospital discharge. Study follow up visits via telephone occurred at 30 days, 6 months, and 12 months from randomisation. Clinical event reporting, EQ-5D-5L and Seattle Angina Questionnaire completion were performed at each of these timepoints.

The GRACE 2.0 score

Previous high-risk subgroup analyses utilised the original GRACE score, with a score of >140 stratifying those patients at highest baseline risk. In the TIMACS and VERDICT trials it was these groups that benefited from an early invasive strategy (9, 15). However, the updated GRACE 2.0 score demonstrates equivalent performance and is easier to implement in clinical practice as compared to the original GRACE Score (23). A notable advantage of GRACE 2.0 is that Killip Class and serum creatinine values are not required for risk calculation. This allows rapid stratification very early during hospital admission once a hs-Tn result is available, thus obviating the need to wait for renal biochemistry results.

Patients at intermediate risk (GRACE 2.0 score ≥90 to <118) were included to attenuate the perceived age bias of the GRACE score, thereby allowing enrolment of younger patients recognised to be at higher risk of future MACE. The following features: anterior ECG changes, ST segment depression, diabetes mellitus on medication, and hs-Tn elevation three times the upper limit of normal, have been demonstrated as determinants of poorer prognosis in N-STEMI (24-26).

RAPID N-STEMI CMR substudy

Imaging studies confined to N-STEMI are few and primarily descriptive, documenting smaller infarct size than in STEMI (27). Novel cardiac magnetic resonance (CMR) markers (such as

For peer review only

myocardial strain and salvage) may add incremental prognostic information to recognised predictors such as left ventricular ejection fraction (LVEF) (28, 29).

The RAPID N-STEMI CMR substudy applied multi-parametric CMR imaging to; 1) assess the impact of the timing of revascularisation on infarct size, volumes and LVEF, myocardial strain, myocardial salvage, and extracellular volume and 2) quantify ischaemic burden post-PCI to ascertain whether this can predict risk of future MACE. The RAPID N-STEMI CMR substudy endpoints are listed in **Table 3**.

Four centres with an established record of high-quality CMR research participated in the substudy. As myocardial injury and infarct size reduces early following MI (30), timing of CMR was standardised and performed at 7 (+/-3) days post admission. This also ensured angiography +/- PCI had been undertaken in both groups, as PCI itself may be associated with further myocardial injury (31, 32). The protocol included cine imaging in long and short axes. Adenosine stress perfusion was performed to assess for residual ischaemic burden and a gadolinium-based contrast agent administered to allow detection of myocardial necrosis and microvascular obstruction (32). Where available, pre- and post-contrast T1 mapping sequences will facilitate estimation of extracellular volume that may indicate more subtle changes in myocardial architecture (33).

All CMR images will be sent to the core laboratory at the National Institute for Health Research Biomedical Research Centre in Leicester for quality control and central analysis, with the interpreting clinicians blinded to patient information and allocated group.

For peer review only

Study endpoints

The RAPID-NSTEMI trial will evaluate the effect of a very early invasive strategy on the binary primary endpoint which is composite of all-cause mortality, non-fatal MI, and admission for heart failure (HF) at 12 months following randomisation. The primary and secondary endpoints are listed in **Table 4.**

Sample size calculation

RAPID N-STEMI is a superiority trial powered to detect a 25% risk reduction in the primary endpoint. With a two-sided test of α=0.05 and 80% power, 964 patients were required in each arm of the study. Assuming up to 5% withdrawal, 5% crossover and 8% requiring coronary artery bypass grafting, 1157 patients were planned to be recruited to each group, resulting in a recruitment target of 2314.

Sample size calculations were based primarily on the subgroup analysis of GRACE >140 high-risk patients in the TIMACS study. The composite endpoint of death, non-fatal MI, and stroke at 6 months occurred in 21.0% of patients in the standard care arm (9). We decided to include admission with HF since there is evidence of this being an important outcome following N-STEMI hospitalisation. Data from *Kaul et al* show that at 12 months following N-STEMI the incidence of admission with HF was 14.8% (34). Based on these data and use of the GRACE 2.0 score, the standard care arm composite event rate of all-cause mortality, non-fatal MI, and admission for HF in RAPID N-STEMI was estimated to be 19% at 12 months.

For peer review only

The CMR substudy is an exploratory mechanistic substudy that had a recruitment target of 200 patients. No formal power calculations were undertaken.

Statistical analysis

A full statistical analysis plan will be completed prior to any analyses being undertaken. Primary analysis will be carried out by intention to treat with imputation for individuals with missing data due to loss to follow-up assessment as having no event. The primary outcome is binary for each individual (yes or no) and therefore participants experiencing more than one of the composite events will only be counted once. The treatment arms will be compared using mixed effects logistic regression, which will be adjusted for randomisation stratification factors hospital site (as a random effect) and GRACE score (as a fixed effect). Treatment comparison estimates will be presented as adjusted odds ratios (OR) and 95% confidence intervals (95% CI).

The analysis of binary secondary outcomes will be carried out in the intention to treat population as per the primary outcome analysis. All other secondary continuous outcomes will be analysed on a complete case approach, where participants will only be included if relevant data are available.

Quality of life data (EQ-5D) will be analysed using a mixed effects model with patient as a random effect to account for repeated measures over time. Each patient will contribute up to four postoperative repeated measures to the model. The model will be adjusted for the stratification variables as above. It is expected that some values at later time points will be

For peer review only

missing. The mixed effects model specified here will include these patients with partially observed data.

Subgroup analyses

An exploratory analysis of the primary outcome in line with the primary analysis plan will be repeated looking for indications of a randomised treatment arm interaction with the following subgroups:

Gender; Female and Male

Age at randomisation: <75 years and ≥75 years

GRACE 2.0 score at admission; >140; >118 & <140; and 90-118

ECG normal vs ECG abnormalities at admission

Exploratory analyses

The primary endpoint will also be analysed as a time-to-first-event outcome. The time will be measured from randomisation and differences between treatment arms compared using Cox's proportional hazards models, with treatment comparisons presented as hazard ratios and 95% confidence interval. All time to event outcomes will be intention to treat with losses to follow-up censored at date last seen.

An exploratory analysis will be conducted repeating the analysis methods of the primary outcome in the efficacy population. The efficacy population excludes individuals that were randomised to Early Intervention not receiving angiography within 12 hours of randomisation OR were randomised to Standard Care receiving angiography within 12 hours (unless participant's procedure performed earlier than anticipated due to clinical deterioration).

For peer review only

The association between CMR outcomes and the primary outcome will be assessed by logistic regression with each CMR variable being included in a separate model. Models will have the clinical outcome as their dependant variable and include the CMR variable as explanatory variable as well as adjusting for treatment arm, site, GRACE score, age and sex.

Funding and sponsor

RAPID N-STEMI is funded by the British Heart Foundation (grant number: CS/17/1/32445). The study sponsor is University Hospitals of Leicester National Health Service (NHS) Trust. The study is registered at ClinicalTrials.gov (NCT03707314).

Ethics and dissemination

The study is conducted in accordance with the principles of the 1996 Helsinki Declarations, International Conference on Harmonisation-Good Clinical Practice (ICH-GCP) guidelines. The trial has been reviewed and approved by the East of England Cambridge East Research Ethics Committee (18/EE/0222). It is anticipated that data completion will be completed by the end of December 2021, and the study results will be submitted for publication within 6 months of completion.

Public and patient involvement

The study was presented to the National Institute for Health Research (NIHR) University of Leicester Biomedical Research Centre (BRC) Patient & Public Involvement Group. Development of the protocol, outcome measures, recruitment to the trial and conduct of the study were discussed. There was a favourable response to the proposed study from the

For peer review only

group. Study progress has been fed back to the Patient & Public Involvement group during the course of the trial. Participants are given a study newsletter when they attend their 12-month clinic visit providing information about the study timelines and when the study results will be known. Access to the findings of the study will be made available in a contemporary and user-friendly way and full details of the results provided if the patient requests them.

Trial coordination

Trial coordination is provided by the Leicester Clinical Trials Unit (LCTU) in collaboration with the Chief Investigator (CI) and the Trial Management Group. LCTU is responsible for overall trial conduct including data management, quality assurance and statistical reporting. LCTU undertook site initiation visits, database training, and ensures all aspects of the trial are performed to the highest ethical and research standards. The study is overseen by a Trial Steering Committee consisting of three experienced clinicians and the CI. An independent Data and Safety Monitoring Board convened to provide independent advice on study conduct and safety issues. Clinical events will be adjudicated by an independent Clinical Events Committee.

Trial progress and impact of the COVID-19 pandemic

In March 2020, non-COVID-19 clinical research in the UK was suspended as NHS staff and resources were repurposed to frontline services in preparation for the volume of COVID-19 patients expected to place severe pressure on the NHS (35). During this time, admissions with N-STEMI declined substantially (36, 37). Following the first wave in the UK, RAPID N-STEMI restarted in late July 2020 at a limited number of sites that had sufficient resource to

For peer review only

recommence recruitment. However, due to the impending second wave of the COVID-19 pandemic the trial was once again suspended in December 2020. Discussions with the funding body took place regarding the strategy for a successful restart, with it agreed an interim pooled event rate (blinded to group allocation) should be calculated. Lower than anticipated event rates were documented and it was agreed with the funder that enrolment should be discontinued for two reasons: 1) the effect of the pandemic on clinical services and 2) the rate of the primary outcome. In summary, 425 (18.4% intended) patients were enrolled to the main trial, with 61 of these participants included in the CMR substudy. The intention is to perform in depth analyses of the available data from these populations and present these in the near future.

Discussion

The optimal timing of revascularisation in higher-risk N-STEMI is a controversial topic, not least because international guidelines that mandate an early (<24 hr) invasive strategy are not supported by prospective randomised-controlled clinical trial data (2, 3). RAPID N-STEMI addresses this knowledge gap. However, like many other clinical trials during the COVID-19 pandemic, RAPID N-STEMI was discontinued due to the emergency restructuring of healthcare and clinical research services. Despite falling short of the recruitment target, RAPID N-STEMI has randomised 425 GRACE score defined higher-risk patients admitted with N-STEMI, making it the third largest study to specifically investigate this important patient population. It will therefore provide an significant contribution to the current evidence base, with dissemination of results planned for 2022.

COVID-19: implications for cardiovascular research

For peer review only

The major challenges faced due to COVID-19 were three-fold. First, significant reductions in admissions with acute coronary syndrome and HF occurred during the pandemic in the UK, with decreases of over to 40% in both disease entities, presumably due to fear of contagion in healthcare settings (36, 38, 39). Not only did this reduce potential research participants, but such declines in admissions become a critical issue for clinical trial event reporting and thus may be a contributory factor to the lower event rates observed in RAPID N-STEMI.

Second, the NHS underwent the largest workforce redeployment since its inception to support severely pressurised frontline services treat the vast numbers of COVID-19 patients attending UK hospitals. Research staff were moved to such clinical areas, resulting in all non-COVID-19 related research being left severely disrupted and placed on hiatus until further notice (40), the ramifications of which are sure to be felt long after the initial effects of the COVID-19 pandemic have abated (41).

Third, and perhaps most fundamentally for RAPID N-STEMI, elective cardiology activity across the UK was effectively cancelled from the beginning of the first wave in March 2020 (35). Suspension of planned cases created greater catheter laboratory capacity for acute MI patients and dramatically reduced the standard of care timing to ICA for N-STEMI in the UK (42). Such changes to catheter laboratory throughput and working patterns resulted in an unmanageable task of ensuring adequate time separation between the very early ICA and standard of care ICA arms in RAPID N-STEMI – essentially the control arm was accelerated. Since any potential differences in clinical outcomes are related to the difference in timing between the trial arms, new systems of care enforced by the COVID-19 pandemic left the

For peer review only

RAPID N-STEMI investigators in a position whereby restarting recruitment would inevitably jeopardise the scientific validity of the trial.

Very early revascularisation in higher-risk N-STEMI: will optimal timing ever be defined?

RAPID N-STEMI is the fifth randomised trial to investigate the timing of an invasive strategy in GRACE score defined higher-risk N-STEMI patients, albeit the first to specifically investigate this higher-risk population. Prior studies report pre-specified GRACE >140 subgroup analyses (**Table 1**). Favourable results were observed in TIMACS and VERDICT (9, 15), while ELISA-3 and RIDDLE N-STEMI showed signals of benefit although were limited by small sample sizes (12, 14). Since clinical event rates are greater in high-risk N-STEMI, it may be expected that an adequately powered study to detect a clinically meaningful difference is achievable. Yet, in recent years outcomes following N-STEMI have improved, largely due to enhanced therapeutics and interventional techniques (43). Therefore, demonstration of superiority for hard clinical endpoints from a very early invasive strategy may not be feasible in the contemporary era because the logistics of delivering an appropriately powered trial may be prohibitive.

The choice of trial endpoints is also of note. The EARLY trial indicated benefit from a very early invasive approach in European Society of Cardiology defined high-risk patients (median GRACE score 122), but such benefit was driven by the softer endpoint of recurrent ischaemic events in a cohort that did not receive P2Y12 inhibitor pre-treatment (17). One may question the clinical relevance of an endpoint, and as such it was not included in the composite primary endpoint of RAPID N-STEMI. Given that practice in many centres is now shifting to a strategy of early ICA in higher-risk N-STEMI patient groups, and that this strategy is now widely

For peer review only

accepted as without excess risk, it appears unlikely that the optimal timing of revascularisation in higher-risk N-STEMI will ever be robustly defined.

Contributorship statement

AsB, MF, CB, NC, ApB, GPM & AHG conceived the idea for the study. AsB, GPM & AHG designed the study protocol. TAK drafted the manuscript. AsB, AL, MF, CPG, JPG, MD, RH, SB, EP, CB, NC, ApB & GPM critically reviewed and approved the final version of the manuscript.

Competing interests

CB is employed by the University of Glasgow which holds consultancy and research agreements for his work with Abbott Vascular, AstraZeneca, Boehringer Ingelheim, Causeway Therapeutics, Coroventis, Genentech, GSK, HeartFlow, Menarini, Neovasc, Siemens Healthcare, and Valo Health. The other co-authors have no relevant disclosures.

Funding

The study is funded by the British Heart Foundation (grant number: CS/17/1/32445). The grant providers were not involved in study design, data acquisition or management, or analysis and writing of final reports.

Acknowledgements

The authors would like to acknowledge the support of the National Institute for Health Research Biomedical Research Centre in Leicester and the Leeds National Institute for Health Research Clinical Research Facility.

For peer review only

References

1. Fox KAA, Clayton TC, Damman P, Pocock SJ, de Winter RJ, Tijssen JGP, et al. Long-term outcome of a routine versus selective invasive strategy in patients with non-ST-segment elevation acute coronary syndrome a meta-analysis of individual patient data. J Am Coll Cardiol. 2010;55(22):2435-45.
2. Collet JP, Thiele H, Barbato E, Barthelemy O, Bauersachs J, Bhatt DL, et al. 2020 ESC Guidelines for the management of acute coronary syndromes in patients presenting without persistent ST-segment elevation. Eur Heart J. 2020.
3. Amsterdam EA, Wenger NK, Brindis RG, Casey DE, Jr., Ganiats TG, Holmes DR, Jr., et al. 2014 AHA/ACC Guideline for the Management of Patients with Non-ST-Elevation Acute Coronary Syndromes: a report of the American College of Cardiology/American Heart Association Task Force on Practice Guidelines. J Am Coll Cardiol. 2014;64(24):e139-e228.
4. Hall M, Bebb OJ, Dondo TB, Yan AT, Goodman SG, Bueno H, et al. Guideline-indicated treatments and diagnostics, GRACE risk score, and survival for non-ST elevation myocardial infarction. Eur Heart J. 2018;39(42):3798-806.
5. van 't Hof AW, de Vries ST, Dambrink JH, Miedema K, Suryapranata H, Hoorntje JC, et al. A comparison of two invasive strategies in patients with non-ST elevation acute coronary syndromes: results of the Early or Late Intervention in unStable Angina (ELISA) pilot study. 2b/3a upstream therapy and acute coronary syndromes. Eur Heart J. 2003;24(15):1401-5.
6. Neumann FJ, Kastrati A, Pogatsa-Murray G, Mehilli J, Bollwein H, Bestehorn HP, et al. Evaluation of prolonged antithrombotic pretreatment ("cooling-off" strategy) before intervention in patients with unstable coronary syndromes: a randomized controlled trial. JAMA. 2003;290(12):1593-9.
7. Montalescot G, Cayla G, Collet JP, Elhadad S, Beygui F, Le Breton H, et al. Immediate vs delayed intervention for acute coronary syndromes: a randomized clinical trial. JAMA. 2009;302(9):947-54.
8. Riezebos RK, Ronner E, Ter Bals E, Slagboom T, Smits PC, ten Berg JM, et al. Immediate versus deferred coronary angioplasty in non-ST-segment elevation acute coronary syndromes. Heart. 2009;95(10):807-12.
9. Mehta SR, Granger CB, Boden WE, Steg PG, Bassand JP, Faxon DP, et al. Early versus delayed invasive intervention in acute coronary syndromes. N Engl J Med. 2009;360(21):2165-75.
10. Sciahbasi A, Madonna M, De Vita M, Agati L, Scioli R, Summaria F, et al. Comparison of immediate vs early invasive strategy in patients with first acute non-ST-elevation myocardial infarction. Clin Cardiol. 2010;33(10):650-5.
11. Thiele H, Rach J, Klein N, Pfeiffer D, Hartmann A, Hambrecht R, et al. Optimal timing of invasive angiography in stable non-ST-elevation myocardial infarction: the Leipzig Immediate versus early and late PercutaneouS coronary Intervention triAl in NSTEMI (LIPSIA-NSTEMI Trial). Eur Heart J. 2012;33(16):2035-43.
12. Badings EA, The SH, Dambrink JH, van Wijngaarden J, Tjeerdsma G, Rasoul S, et al. Early or late intervention in high-risk non-ST-elevation acute coronary syndromes: results of the ELISA-3 trial. EuroIntervention. 2013;9(1):54-61.
13. Reuter PG, Rouchy C, Cattan S, Benamer H, Jullien T, Beruben A, et al. Early invasive strategy in high-risk acute coronary syndrome without ST-segment elevation. The Sisca randomized trial. Int J Cardiol. 2015;182:414-8.

For peer review only

14. Milosevic A, Vasiljevic-Pokrajcic Z, Milasinovic D, Marinkovic J, Vukcevic V, Stefanovic B, et al. Immediate Versus Delayed Invasive Intervention for Non-STEMI Patients: The RIDDLE-NSTEMI Study. JACC Cardiovasc Interv. 2016;9(6):541-9.
15. Kofoed KF, Kelbaek H, Hansen PR, Torp-Pedersen C, Hofsten D, Klovgaard L, et al. Early Versus Standard Care Invasive Examination and Treatment of Patients With Non-ST-Segment Elevation Acute Coronary Syndrome. Circulation. 2018;138(24):2741-50.
16. Rasmussen MB, Stengaard C, Sorensen JT, Riddervold IS, Sondergaard HM, Niemann T, et al. Comparison of Acute Versus Subacute Coronary Angiography in Patients With NON-ST-Elevation Myocardial Infarction (from the NONSTEMI Trial). Am J Cardiol. 2019;124(6):825-32.
17. Lemesle G, Laine M, Pankert M, Boueri Z, Motreff P, Paganelli F, et al. Optimal Timing of Intervention in NSTE-ACS Without Pre-Treatment: The EARLY Randomized Trial. JACC Cardiovasc Interv. 2020;13(8):907-17.
18. Fagel ND, Amoroso G, Vink MA, Slagboom T, van der Schaaf RJ, Herrman JP, et al. An immediate or early invasive strategy in non-ST-elevation acute coronary syndrome: The OPTIMA-2 randomized controlled trial. Am Heart J. 2021;234:42-50.
19. Jobs A, Mehta SR, Montalescot G, Vicaut E, Van't Hof AWJ, Badings EA, et al. Optimal timing of an invasive strategy in patients with non-ST-elevation acute coronary syndrome: a meta-analysis of randomised trials. Lancet. 2017;390(10096):737-46.
20. Neumann FJ, Sousa-Uva M, Ahlsson A, Alfonso F, Banning AP, Benedetto U, et al. 2018 ESC/EACTS Guidelines on myocardial revascularization. Eur Heart J. 2019;40(2):87-165.
21. Herdman M, Gudex C, Lloyd A, Janssen M, Kind P, Parkin D, et al. Development and preliminary testing of the new five-level version of EQ-5D (EQ-5D-5L). Qual Life Res. 2011;20(10):1727-36.
22. Spertus JA, Winder JA, Dewhurst TA, Deyo RA, Prodzinski J, McDonell M, et al. Development and evaluation of the Seattle Angina Questionnaire: a new functional status measure for coronary artery disease. J Am Coll Cardiol. 1995;25(2):333-41.
23. Fox KA, Fitzgerald G, Puymirat E, Huang W, Carruthers K, Simon T, et al. Should patients with acute coronary disease be stratified for management according to their risk? Derivation, external validation and outcomes using the updated GRACE risk score. BMJ Open. 2014;4(2):e004425.
24. Stone PH, Raabe DS, Jaffe AS, Gustafson N, Muller JE, Turi ZG, et al. Prognostic significance of location and type of myocardial infarction: independent adverse outcome associated with anterior location. J Am Coll Cardiol. 1988;11(3):453-63.
25. Mak KH, Topol EJ. Emerging concepts in the management of acute myocardial infarction in patients with diabetes mellitus. J Am Coll Cardiol. 2000;35(3):563-8.
26. Bagai A, Huang Z, Lokhnygina Y, Harrington RA, Armstrong PW, Strony J, et al. Magnitude of troponin elevation and long-term clinical outcomes in acute coronary syndrome patients treated with and without revascularization. Circ Cardiovasc Interv. 2015;8(6):e002314.
27. Plein S, Younger JF, Sparrow P, Ridgway JP, Ball SG, Greenwood JP. Cardiovascular magnetic resonance of scar and ischemia burden early after acute ST elevation and non-ST elevation myocardial infarction. J Cardiovasc Magn Reson. 2008;10:47.
28. Eitel I, Stiermaier T, Lange T, Rommel KP, Koschalka A, Kowallick JT, et al. Cardiac Magnetic Resonance Myocardial Feature Tracking for Optimized Prediction of Cardiovascular Events Following Myocardial Infarction. JACC Cardiovasc Imaging. 2018;11(10):1433-44.

For peer review only

29. Eitel I, Desch S, Fuernau G, Hildebrand L, Gutberlet M, Schuler G, et al. Prognostic significance and determinants of myocardial salvage assessed by cardiovascular magnetic resonance in acute reperfused myocardial infarction. J Am Coll Cardiol. 2010;55(22):2470-9.
30. Mather AN, Fairbairn TA, Artis NJ, Greenwood JP. Timing of Cardiovascular MR Imaging after Acute Myocardial Infarction : Effect on Estimates of Infarct Characteristics and Prediction of Late Ventricular Remodeling. Radiology. 2011;261:116-26.
31. Selvanayagam JB, Porto I, Channon K, Petersen SE, Francis JM, Neubauer S, et al. Troponin elevation after percutaneous coronary intervention directly represents the extent of irreversible myocardial injury: insights from cardiovascular magnetic resonance imaging. Circulation. 2005;111(8):1027-32.
32. McCann GP, Khan JN, Greenwood JP, Nazir S, Dalby M, Curzen N, et al. Complete Versus Lesion-Only Primary PCI: The Randomized Cardiovascular MR CvLPRIT Substudy. J Am Coll Cardiol. 2015;66(24):2713-24.
33. Singh A, Greenwood JP, Berry C, Dawson DK, Hogrefe K, Kelly DJ, et al. Comparison of exercise testing and CMR measured myocardial perfusion reserve for predicting outcome in asymptomatic aortic stenosis: the PRognostic Importance of MIcrovascular Dysfunction in Aortic Stenosis (PRIMID AS) Study. Eur Heart J. 2017;38(16):1222-9.
34. Kaul P, Ezekowitz JA, Armstrong PW, Leung BK, Savu A, Welsh RC, et al. Incidence of heart failure and mortality after acute coronary syndromes. Am Heart J. 2013;165(3):385.e2.
35. NHS England. Next steps on NHS response to COVID-19: Letter from Sir Simon Stevens and Amanda Pritchard 2020 [Available from: https://www.england.nhs.uk/coronavirus/wp-content/uploads/sites/52/2020/03/20200317-NHS-COVID-letter-FINAL.pdf.
36. Mafham MM, Spata E, Goldacre R, Gair D, Curnow P, Bray M, et al. COVID-19 pandemic and admission rates for and management of acute coronary syndromes in England. Lancet. 2020.
37. Wu J, Mamas M, Rashid M, Weston C, Hains J, Luescher T, et al. Patient response, treatments and mortality for acute myocardial infarction during the COVID-19 pandemic. Eur Heart J Qual Care Clin Outcomes. 2020.
38. Shoaib A, Van Spall HGC, Wu J, Cleland JGF, McDonagh TA, Rashid M, et al. Substantial decline in hospital admissions for heart failure accompanied by increased community mortality during COVID-19 pandemic. Eur Heart J Qual Care Clin Outcomes. 2021.
39. Wu J, Mamas MA, de Belder MA, Deanfield JE, Gale CP. Second Decline in Admissions With Heart Failure and Myocardial Infarction During the COVID-19 Pandemic. J Am Coll Cardiol. 2021;77(8):1141-3.
40. National Institute for Health Research. DHSC issues guidance on the impact of COVID-19 on research funded or supported by NIHR, updated 19 Mar 2020 [Available from: https://www.nihr.ac.uk/news/dhsc-issues-guidance-on-the-impact-on-covid-19-on-research-funded-or-supported-by-nihr/24469.
41. van Dorn A. COVID-19 and readjusting clinical trials. Lancet. 2020;396(10250):523-4.
42. Kwok CS, Gale CP, Curzen N, de Belder MA, Ludman P, Luscher TF, et al. Impact of the COVID-19 Pandemic on Percutaneous Coronary Intervention in England: Insights From the British Cardiovascular Intervention Society PCI Database Cohort. Circ Cardiovasc Interv. 2020;13(11):e009654.
43. Hall M, Dondo TB, Yan AT, Goodman SG, Bueno H, Chew DP, et al. Association of Clinical Factors and Therapeutic Strategies With Improvements in Survival Following Non-ST-Elevation Myocardial Infarction, 2003-2013. JAMA. 2016;316(10):1073-82.

Figure Legends

Figure 1: RAPID N-STEMI study flow diagram

CMR: cardiac magnetic resonance; ECG: electrocardiogram; hs-Tn: high sensitivity troponin N-STEMI: non-ST elevation myocardial infarction; OMT: optimal medical therapy; SAQ: Seattle Angina Questionnaire; STEMI: ST elevation myocardial infarction; ULN: upper limit of normal

For peer review only

Table 1: Higher-risk N-STEMI (GRACE score >140) subgroup analyses from randomised studies comparing early and delayed invasive strategies

Trial	Patients	Time to ICA: early (median & IQR, h)	Time to ICA: delayed (median & IQR, h)	Primary outcome	Results
TIMACS 2009	961	14.0 (3.0 – 21.0)	50.0 (41.0 – 81.0)	6-month death, non-fatal MI, stroke	Early = 13.9%<br>Delayed = 21.0%<br>HR 0.65, 95% CI 0.48-0.89, p = 0.006
ELISA-3 2013	224	2.6 (1.2 – 6.2)	54.9 (44.2 – 74.5)	30-day death, non-fatal MI, recurrent ischaemia	Early = 10.5%<br>Delayed = 19.1%<br>HR 0.55, 95% CI 0.29-1.10, p=0.26
RIDDLE-NSTEMI 2016	123	1.4 (1.0 – 2.2)	61.0 (35.8 – 85.0)	30-day death, non-fatal MI	Early = 10.7%<br>Delayed = 17.9%<br>HR 0.56, 95% CI 0.21-1.51 p=0.12
VERDICT 2018	1025	4.7 (3.0 – 12.2)	61.6 (39.4 – 87.8)	Death, non-fatal MI, refractory ischaemia, admission for heart failure at median 4.3 years	Early = 34.0%<br>Delayed = 40.1%<br>HR 0.81, 95% CI 0.66-0.99, p = 0.023

CI: confidence interval; HR: hazard ratio; ICA: invasive coronary angiography; IQR: interquartile range; MI: myocardial infarction

For peer review only

Table 2: RAPID N-STEMI inclusion and exclusion criteria

Inclusion criteria	Exclusion criteria
>18 years of age	ST elevation myocardial infarction
Clinical diagnosis of N-STEMI comprising:<br>▪ Cardiovascular symptoms suggestive of myocardial ischaemia<br>▪ Elevated high-sensitivity troponin I or T	Evident type 2 myocardial infarction
Symptoms <12 hours prior to admission	Previous known cardiomyopathy
GRACE 2.0 score ≥118 or if GRACE 2.0 score ≥90 but <118 must have at least one high-risk feature:<br>▪ Anterior location of ECG changes (V2-V5)<br>▪ ST segment depression in 2 contiguous leads of 0.15mV/1.5mm<br>▪ Diabetes mellitus on medication<br>▪ Elevated high-sensitivity troponin 3x upper limit of normal	Need for urgent PCI according to ESC Guidelines (haemodynamic instability, VT, VF, recurrent or persistent pain)
Intention to perform angiography and, if indicated, follow-on revascularisation	Cardiogenic shock
Provision of verbal assent followed by written informed consent	Severe valvular heart disease
	Any contraindication to PCI
	Current participation in another intervention trial

ECG: electrocardiogram; ESC: European Society of Cardiology; GRACE: Global Registry of Acute Coronary Events; N-STEMI: non-ST elevation myocardial infarction; PCI: percutaneous coronary intervention; VF: ventricular fibrillation; VT: ventricular tachycardia

Table 3: RAPID N-STEMI CMR study endpoints

Primary outcome	Secondary outcomes
Infarct size (% left ventricular mass)	Left ventricular volumes and ejection fraction
	Myocardial salvage index
	Extracellular volume
	Ischaemic burden
	Global myocardial strain

For peer review only

Table 4: RAPID N-STEMI study endpoints

Primary outcome	Secondary outcomes
All-cause mortality, non-fatal myocardial infarction, and admission for heart failure at 12 months	Individual components of primary composite outcome
	Cardiovascular mortality
	Ischaemia-driven revascularisation
	BARC 3-5 major bleeding
	Stroke
	Length of inpatient stay
	Admission for any cause
	Events prior to angiography
	Quality of life (Seattle Angina Questionnaire and EQ-5D-5L questionnaires)
	Cost-efficacy analysis
	Proportion of patients requiring emergency revascularisation in group B
	Total VARC-2 classified access site complications
	Major VARC-2 classified access site complications

BARC: Bleeding Academic Research Consortium; VARC: Vascular Access Research Consortium

For peer review only

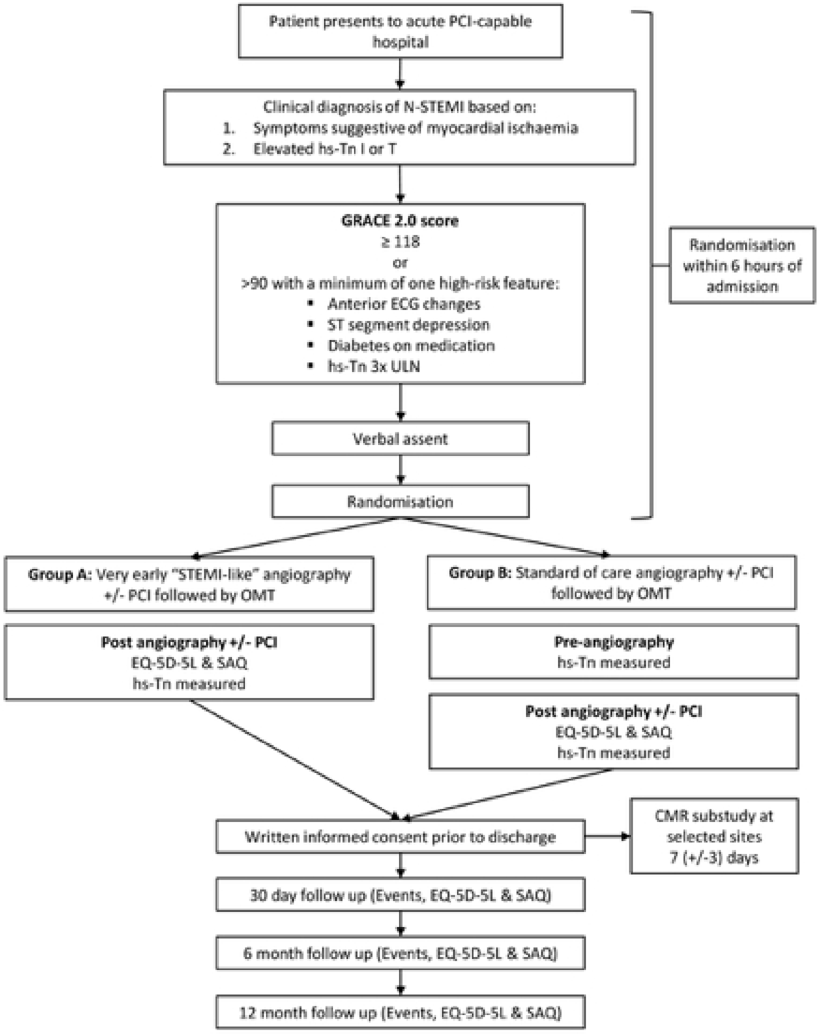

Figure 1: RAPID N-STEMI study flow diagram

CMR: cardiac magnetic resonance; ECG: electrocardiogram; hs-Tn: high sensitivity troponin N-STEMI: non-ST elevation myocardial infarction; OMT: optimal medical therapy; SAQ: Seattle Angina Questionnaire; STEMI: ST elevation myocardial infarction; ULN: upper limit of normal

38x54mm (300 x 300 DPI)

Reporting checklist for protocol of a clinical trial.

Based on the SPIRIT guidelines.

Instructions to authors

Complete this checklist by entering the page numbers from your manuscript where readers will find each of the items listed below.

Your article may not currently address all the items on the checklist. Please modify your text to include the missing information. If you are certain that an item does not apply, please write "n/a" and provide a short explanation.

Upload your completed checklist as an extra file when you submit to a journal.

In your methods section, say that you used the SPIRITreporting guidelines, and cite them as:

Chan A-W, Tetzlaff JM, Gøtzsche PC, Altman DG, Mann H, Berlin J, Dickersin K, Hróbjartsson A, Schulz KF, Parulekar WR, Krleža-Jerić K, Laupacis A, Moher D. SPIRIT 2013 Explanation and Elaboration: Guidance for protocols of clinical trials. BMJ. 2013;346:e7586

		Reporting Item	Page Number
**Administrative information**			
Title	#1	Descriptive title identifying the study design, population, interventions, and, if applicable, trial acronym	1

For peer review only

Trial registration	#2a	Trial identifier and registry name. If not yet registered, name of intended registry	2
Trial registration: data set	#2b	All items from the World Health Organization Trial Registration Data Set	2
Protocol version	#3	Date and version identifier	3
Funding	#4	Sources and types of financial, material, and other support	20
Roles and responsibilities: contributorship	#5a	Names, affiliations, and roles of protocol contributors	20
Roles and responsibilities: sponsor contact information	#5b	Name and contact information for the trial sponsor	8
Roles and responsibilities: sponsor and funder	#5c	Role of study sponsor and funders, if any, in study design; collection, management, analysis, and interpretation of data; writing of the report; and the decision to submit the report for publication, including whether they will have ultimate authority over any of these activities	20
Roles and responsibilities: committees	#5d	Composition, roles, and responsibilities of the coordinating centre, steering committee, endpoint adjudication committee, data management team, and	11

For peer review only

		other individuals or groups overseeing the trial, if applicable (see Item 21a for data monitoring committee)	
**Introduction**			
Background and rationale	#6a	Description of research question and justification for undertaking the trial, including summary of relevant studies (published and unpublished) examining benefits and harms for each intervention	5
Background and rationale: choice of comparators	#6b	Explanation for choice of comparators	5
Objectives	#7	Specific objectives or hypotheses	6
Trial design	#8	Description of trial design including type of trial (eg, parallel group, crossover, factorial, single group), allocation ratio, and framework (eg, superiority, equivalence, non-inferiority, exploratory)	6
**Methods: Participants, interventions, and outcomes**			
Study setting	#9	Description of study settings (eg, community clinic, academic hospital) and list of countries where data will be collected. Reference to where list of study sites can be obtained	6

For peer review only

Eligibility criteria	#10	Inclusion and exclusion criteria for participants. If applicable, eligibility criteria for study centres and individuals who will perform the interventions (eg, surgeons, psychotherapists)	7
Interventions: description	#11a	Interventions for each group with sufficient detail to allow replication, including how and when they will be administered	7
Interventions: modifications	#11b	Criteria for discontinuing or modifying allocated interventions for a given trial participant (eg, drug dose change in response to harms, participant request, or improving / worsening disease)	7
Interventions: adherance	#11c	Strategies to improve adherence to intervention protocols, and any procedures for monitoring adherence (eg, drug tablet return; laboratory tests)	7
Interventions: concomitant care	#11d	Relevant concomitant care and interventions that are permitted or prohibited during the trial	7
Outcomes	#12	Primary, secondary, and other outcomes, including the specific measurement variable (eg, systolic blood pressure), analysis metric (eg, change from baseline, final value, time to event), method of aggregation (eg, median, proportion), and time point for each outcome. Explanation of the clinical relevance of chosen efficacy and harm outcomes is strongly recommended	10

For peer review only

Participant timeline	#13	Time schedule of enrolment, interventions (including any run-ins and washouts), assessments, and visits for participants. A schematic diagram is highly recommended (see Figure)	10
Sample size	#14	Estimated number of participants needed to achieve study objectives and how it was determined, including clinical and statistical assumptions supporting any sample size calculations	10
Recruitment	#15	Strategies for achieving adequate participant enrolment to reach target sample size	10
**Methods: Assignment of interventions (for controlled trials)**			
Allocation: sequence generation	#16a	Method of generating the allocation sequence (eg, computer-generated random numbers), and list of any factors for stratification. To reduce predictability of a random sequence, details of any planned restriction (eg, blocking) should be provided in a separate document that is unavailable to those who enrol participants or assign interventions	7
Allocation concealment mechanism	#16b	Mechanism of implementing the allocation sequence (eg, central telephone; sequentially numbered, opaque,	7

For peer review only

		sealed envelopes), describing any steps to conceal the sequence until interventions are assigned	
Allocation: implementation	#16c	Who will generate the allocation sequence, who will enrol participants, and who will assign participants to interventions	7
Blinding (masking)	#17a	Who will be blinded after assignment to interventions (eg, trial participants, care providers, outcome assessors, data analysts), and how	7
Blinding (masking): emergency unblinding	#17b	If blinded, circumstances under which unblinding is permissible, and procedure for revealing a participant's allocated intervention during the trial	7
**Methods: Data collection, management, and analysis**			
Data collection plan	#18a	Plans for assessment and collection of outcome, baseline, and other trial data, including any related processes to promote data quality (eg, duplicate measurements, training of assessors) and a description of study instruments (eg, questionnaires, laboratory tests) along with their reliability and validity, if known. Reference to where data collection forms can be found, if not in the protocol	8

For peer review only

Data collection plan: retention	#18b	Plans to promote participant retention and complete follow-up, including list of any outcome data to be collected for participants who discontinue or deviate from intervention protocols	8
Data management	#19	Plans for data entry, coding, security, and storage, including any related processes to promote data quality (eg, double data entry; range checks for data values). Reference to where details of data management procedures can be found, if not in the protocol	8
Statistics: outcomes	#20a	Statistical methods for analysing primary and secondary outcomes. Reference to where other details of the statistical analysis plan can be found, if not in the protocol	11
Statistics: additional analyses	#20b	Methods for any additional analyses (eg, subgroup and adjusted analyses)	11
Statistics: analysis population and missing data	#20c	Definition of analysis population relating to protocol non-adherence (eg, as randomised analysis), and any statistical methods to handle missing data (eg, multiple imputation)	11
**Methods: Monitoring**			
Data monitoring: formal committee	#21a	Composition of data monitoring committee (DMC); summary of its role and reporting structure; statement of whether it is independent from the sponsor and competing interests; and reference to where further	11

For peer review only

		details about its charter can be found, if not in the protocol. Alternatively, an explanation of why a DMC is not needed	
Data monitoring: interim analysis	#21b	Description of any interim analyses and stopping guidelines, including who will have access to these interim results and make the final decision to terminate the trial	n/a
Harms	#22	Plans for collecting, assessing, reporting, and managing solicited and spontaneously reported adverse events and other unintended effects of trial interventions or trial conduct	n/a
Auditing	#23	Frequency and procedures for auditing trial conduct, if any, and whether the process will be independent from investigators and the sponsor	n/a
**Ethics and dissemination**			
Research ethics approval	#24	Plans for seeking research ethics committee / institutional review board (REC / IRB) approval	8
Protocol amendments	#25	Plans for communicating important protocol modifications (eg, changes to eligibility criteria, outcomes, analyses) to relevant parties (eg, investigators, REC / IRBs, trial participants, trial registries, journals, regulators)	n/a

For peer review only

Consent or assent	#26a	Who will obtain informed consent or assent from potential trial participants or authorised surrogates, and how (see Item 32)	n/a
Consent or assent: ancillary studies	#26b	Additional consent provisions for collection and use of participant data and biological specimens in ancillary studies, if applicable	n/a
Confidentiality	#27	How personal information about potential and enrolled participants will be collected, shared, and maintained in order to protect confidentiality before, during, and after the trial	n/a
Declaration of interests	#28	Financial and other competing interests for principal investigators for the overall trial and each study site	20
Data access	#29	Statement of who will have access to the final trial dataset, and disclosure of contractual agreements that limit such access for investigators	21
Ancillary and post trial care	#30	Provisions, if any, for ancillary and post-trial care, and for compensation to those who suffer harm from trial participation	n/a
Dissemination policy: trial results	#31a	Plans for investigators and sponsor to communicate trial results to participants, healthcare professionals, the public, and other relevant groups (eg, via publication, reporting in results databases, or other data sharing arrangements), including any publication restrictions	20

For peer review only

Dissemination policy: authorship	#31b	Authorship eligibility guidelines and any intended use of professional writers	n/a
Dissemination policy: reproducible research	#31c	Plans, if any, for granting public access to the full protocol, participant-level dataset, and statistical code	20

Appendices

Informed consent materials	#32	Model consent form and other related documentation given to participants and authorised surrogates	n/a
Biological specimens	#33	Plans for collection, laboratory evaluation, and storage of biological specimens for genetic or molecular analysis in the current trial and for future use in ancillary studies, if applicable	n/a

The SPIRIT Explanation and Elaboration paper is distributed under the terms of the Creative Commons Attribution License CC-BY-NC. This checklist was completed on 26. July 2021 using https://www.goodreports.org/, a tool made by the EQUATOR Network in collaboration with Penelope.ai

For peer review only
